# Discovery of a rapidly evolving yeast defense factor, *KTD1*, against the secreted killer toxin K28

**DOI:** 10.1073/pnas.2217194120

**Published:** 2023-02-17

**Authors:** Ilya Andreev, Kamilla M. E. Laidlaw, Simone M. Giovanetti, Guillaume Urtecho, Daniel Shriner, Joshua S. Bloom, Chris MacDonald, Meru J. Sadhu

**Affiliations:** ^a^Computational and Statistical Genomics Branch, National Human Genome Research Institute, NIH, Bethesda, MD 20892; ^b^Biology Department, University of York, York YO10 5DD, UK; ^c^York Biomedical Research Institute, University of York, York YO10 5NG, UK; ^d^Molecular Biology Interdepartmental Doctoral Program, University of California, Los Angeles, CA 90095; ^e^Center for Research on Genomics and Global Health, National Human Genome Research Institute, NIH, Bethesda, MD 20892; ^f^Department of Human Genetics, University of California, Los Angeles, CA 90095; ^g^Department of Biological Chemistry, University of California, Los Angeles, CA 90095; ^h^HHMI, University of California, Los Angeles, CA 90095; ^i^Institute for Quantitative and Computational Biology, University of California, Los Angeles, CA 90095; ^j^Department of Computational Medicine, University of California, Los Angeles, CA 90095

**Keywords:** genetics, evolution, host defense, killer yeast, quantitative genetics

## Abstract

Secreted protein toxins are widely used weapons in conflicts between organisms. Elucidating how organisms genetically adapt to defend themselves against these toxins is fundamental to understanding the coevolutionary dynamics of competing organisms. Within yeast communities, “killer” toxins are secreted to kill nearby sensitive yeast, providing a fitness advantage in competitive growth environments. Natural yeast isolates vary in their sensitivity to these toxins, but to date, no polymorphic genetic factors contributing to defense have been identified. We investigated the variation in resistance to the killer toxin K28 across diverse natural isolates of the *Saccharomyces cerevisiae* population. Using large-scale linkage mapping, we discovered a novel defense factor, which we named *KTD1*. We identified many *KTD1* alleles, which provided different levels of K28 resistance. *KTD1* is a member of the DUP240 gene family of unknown function, which is rapidly evolving in a region spanning its two encoded transmembrane helices. We found that this domain is critical to *KTD1*’s protective ability. Our findings implicate *KTD1* as a key polymorphic factor in the defense against K28 toxin.

## Significance

Protein toxins are used throughout biology as weapons in evolutionary conflicts. A fascinating example is killer yeast, which secrete virally encoded killer toxins that inhibit the growth of susceptible yeast strains. Yeast in nature vary in their sensitivity to killer toxins, but to date, the basis of this variation has not been understood. We discovered that the budding yeast *Saccharomyces cerevisiae* has a rapidly evolving defense factor against the killer toxin K28, which we name *KTD1*. *KTD1* is a member of the DUP240 gene family. Although the yeast genome has many DUP240 genes, their cellular function has been enigmatic. Our results uncover the role of a DUP240 gene in defense against killer toxin as part of an evolutionary conflict.

Conflict between organisms is a major evolutionary force, both within and between species, and can lead to complex molecular arms races of competing evolutionary adaptation. Secreted protein toxins are a common weapon in biological conflict, encountered across all domains of life (1–3). For instance, deadly protein toxins for humans include cholera toxin and anthrax toxin from bacteria, ricin from plants, and snake and spider venom toxins from animals. Protein toxins have various features that can make them extremely potent: For instance, they can evolve to bind their targets with very high binding affinity, and many of them can iterate toxic effects through enzymatic activity. Exposure to protein toxins can select for adaptations in targeted organisms that allow them to resist the toxic effects. Such adaptations can occur through changes to the targeted protein to block the toxin from binding (4–10); however, the need to maintain the protein’s function constrains the available mutational space. An alternate strategy that allows for greater adaptability is to develop defense factors, such as mammalian pyrin, which stimulates cytokine production and programmed cell death upon detecting bacterial toxin-mediated inhibition of the highly conserved RhoA GTPase (11).

Naturally occurring toxin-secreting strains of *Saccharomyces cerevisiae*, termed killer yeast, are a remarkable example of conflict in the microbial world (12). They are infected by double-stranded RNA killer viruses, which encode killer toxins that the infected yeast secrete to inhibit the growth of nearby susceptible yeast. Growing colonies of infected yeast on lawns of susceptible yeast creates striking halos around the infected colonies. In contrast to the examples above, the toxin-secreting cells can even be members of the same species as the targeted cells, as the virus provides self-immunity against the secreted toxins. Thus, the secretion of toxins is beneficial to the host in competition with sensitive uninfected cells (13–15). Many fungal killer viruses have been discovered, exerting their lethal effects through a variety of mechanisms, including membrane permeabilization (12) and transfer RNA (tRNA) cleavage (16). Some killer toxins kill cells after gaining access to their cytoplasm, a strategy commonly employed by protein toxins, including cholera toxin, anthrax lethal toxin, and ricin (17). The killer toxin K28, encoded by the M28 virus, enters target cells after binding to mannoproteins on the outer surface of the cell wall. The exact mechanism of K28 entry into the cell is unclear, although clathrin-mediated endocytosis is known to be required, in addition to other endocytic trafficking factors (18, 19). Following endocytosis, internalized K28 avoids vacuolar degradation through an unknown mechanism, instead hijacking the retrograde trafficking pathway to get to the endoplasmic reticulum via the Golgi apparatus, subsequently translocating to the cytoplasm and ultimately the nucleus, and inducing G1/S cell cycle arrest (20).

In this study, we were interested in understanding whether cells have developed protection mechanisms against K28 toxicity. Population studies have shown that susceptibility to K28 varies between and within yeast species (21–23), which could indicate adaptation to K28 toxicity. To date, the genetic factors that underlie this variation have not been identified, which would be fundamental to understanding whether, and how, yeast resist K28 toxicity. To investigate the evolution of naturally occurring resistance to K28, we performed a linkage study between yeast strains that naturally vary in K28 susceptibility.

## Results

### Natural Variation in K28 Resistance among Yeast Isolates.

**Fig. 1.**
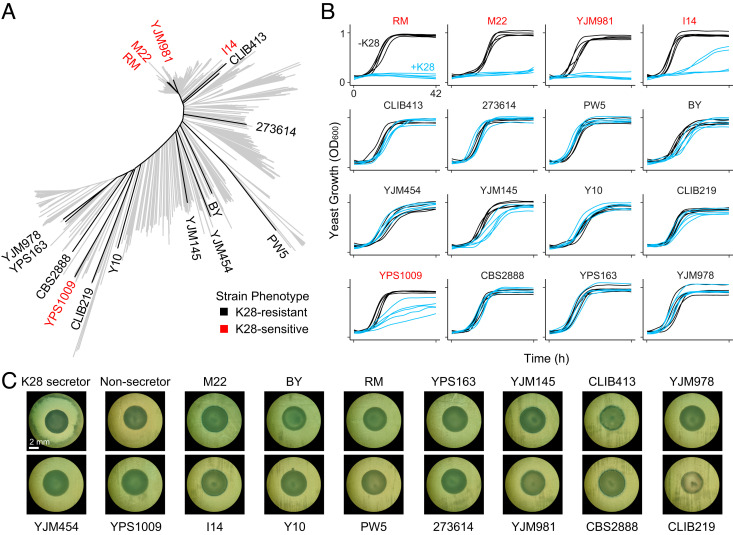
High natural variation in K28 resistance. (*A*) Neighbor-joining phylogenetic tree of 1,011 budding yeast isolates (28), highlighting the 16 strains selected for our panel. Name coloring reflects K28 toxin resistance, with red labels for sensitive strains and black labels for resistant strains, as determined in part B. (*B*) Growth curves of 16 diverse *S. cerevisiae* strains in minimal media with or without K28 (blue and black, respectively; *n* = 5). +K28 and −K28 media were generated from supernatant prepared from a diploid toxin-secreting strain and a virus-cured derivative of that strain, respectively. K28 resistance was quantified as the ratio of the areas under the growth curves in media containing or lacking K28 (AUC_+K28_ / AUC_−K28_) (*SI Appendix*, Fig. S1). Statistical analysis was performed using one-way ANOVA followed by Tukey's post hoc Honest Significant Difference (HSD) test, identifying five strains as K28-sensitive (strain names in red) in comparisons with BY (*P* adjusted < 6.6 × 10^−6^). (*C*) The 16-isolate panel was tested for production of K28 toxin by whether they killed the hypersensitive yeast strain 192.2d. Spots of each strain were grown on a lawn of 192.2d cells; strains producing K28 will generate a halo of cleared 192.2d cells (19). An M28-infected strain with a hypersecretion phenotype due to *SKI2* loss of function (“K28 secretor,” MSY52) was spotted as a positive control, and a virus-cured derivative of that strain (“Non-secretor,” MSY53) was spotted as a negative control. Each spot shown is a representative of three replicate spottings; all replicates are shown in *SI Appendix*, Fig. S2.

We surveyed K28 toxin resistance in a diverse panel of 16 *S. cerevisiae* strains isolated from a variety of geographical locations and ecological settings (24) (Fig. 1*A* and *SI Appendix*, Table S1) and found that they ranged from completely resistant to highly sensitive to K28 (Fig. 1*B* and *SI Appendix*, Fig. S1), consistent with past reports of variation in killer toxin resistance (21–23). Yeast infected with M28 virus are resistant to K28, as killer viruses confer toxin self-immunity upon the host cell (25). However, we did not detect M28 virus in any of these strains (Fig. 1*C* and *SI Appendix*, Figs. S2 and S3), in keeping with previous findings that most yeast isolates are not infected by killer viruses (22, 23, 26, 27). This suggests that differences in toxin resistance are due to variation in chromosomally encoded genetic factors.

### Identification of a Novel Polymorphic Defense Factor, KTD1.

**Fig. 2.**
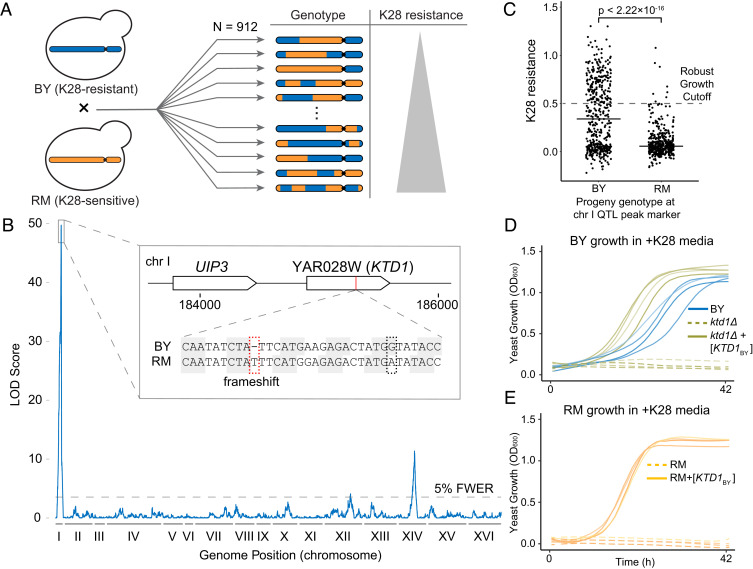
QTL mapping reveals YAR028W (*KTD1*) as a major genetic factor in K28 toxin resistance. (*A*) Schematic representation of the workflow leading up to QTL mapping. 912 recombinant haploid progeny from a cross between BY and RM were assayed for K28 resistance, measured as AUC_+K28_ / AUC_−K28_. (*B*) QTL mapping results. Shown is the degree of association of a genomic locus with K28 resistance phenotype, measured as the LOD score, plotted against genomic coordinates. Three significant QTLs were found above the 5% FWER significance threshold (LOD = 3.55, gray dashed line). The *Inset* shows the genomic context of the QTL peak on chromosome I. (*C*) Phenotypic distribution of 912 BY × RM progeny grouped by their genotype at the peak marker for the chr I QTL. *P* < 2.22 × 10^−16^ by Welch’s two-sample *t* test. The solid horizontal black lines denote mean phenotypes. We used an AUC_+K28_/AUC_−K28_ cutoff of 0.5 to define robust growth. (*D*) Growth of BY with an empty vector, and of BY *yar028wΔ* (*ktd1Δ*) carrying either an empty vector or YAR028W_BY_. Strains were grown in K28-containing media. Four biological replicates are shown in each plot. (*E*) Growth of RM carrying either an empty vector or the YAR028W_BY_ (*KTD1*_BY_) allele. In *D* and *E*, strains with YAR028W_BY_ (*KTD1*_BY_) were significantly more K28-resistant than those without, *P* < 0.005 by Welch’s two-sample one-tailed *t* test (*SI Appendix*, Fig. S6).

To identify genes controlling variation in toxin resistance, we initially focused on the K28-resistant laboratory isolate BY and the highly sensitive wine isolate RM (Fig. 1*B* and *SI Appendix*, Table S1). We determined the toxin resistance of 912 fully genotyped haploid segregants generated from a BY × RM cross (29) by comparing growth in media with and without K28 toxin (Fig. 2*A*). Linkage analysis revealed three regions of the genome (quantitative trait loci, or QTLs) where genetic differences between BY and RM caused differences in K28 resistance (Fig. 2*B* and *SI Appendix*, Table S2). The strongest QTL was on chromosome I: Only 2% of segregants with the RM allele at this locus grew robustly in the presence of K28, compared to 40% of the segregants with the BY allele (Fig. 2*C*). This QTL spanned 4.0 kb (95% confidence interval), with the peak located between the genes *UIP3* and YAR028W. Both genes are members of the DUP240 gene family of unknown function (30, 31).

We examined *UIP3* and YAR028W. The RM allele of YAR028W, YAR028W_RM_, had a 1-nucleotide frameshift mutation at codon 136 (Fig. 2 *B*, *Inset*), leading to 13 additional out-of-frame codons and a premature stop; in comparison, the BY allele of YAR028W is intact and 235 codons long. In contrast, both strains had full-length *UIP3* alleles. Analysis of existing ribosome footprinting data (32) showed that YAR028W is expressed and translated in BY, but its translation ends prematurely in RM, near the frameshift mutation (*SI Appendix*, Fig. S4). Furthermore, a genome-wide screen of 4,806 deletion mutants in the yeast knockout collection, which is isogenic to BY, annotated the YAR028W deletion mutant, *yar028wΔ,* as partially hypersensitive to K28 (19). We confirmed that *yar028wΔ* is sensitive to K28, whereas we observed no effect of deleting *UIP3* (Fig. 2*D* and *SI Appendix*, Figs. S5 and S6). Importantly, we expressed the BY and RM alleles of YAR028W from a centromeric plasmid under their native promoters and found that the BY allele of YAR028W*,* YAR028W_BY_, provided strong protection against K28 in the sensitive RM and *yar028wΔ* strains (Fig. 2 *D* and *E* and *SI Appendix*, Fig. S6), while the RM allele had no effect (*SI Appendix*, Fig. S6). We concluded that YAR028W underlies the dramatic difference in K28 resistance between BY and RM. Therefore, we designated YAR028W as *KTD1* (*k*iller *t*oxin *d*efense).

**Fig. 3.**
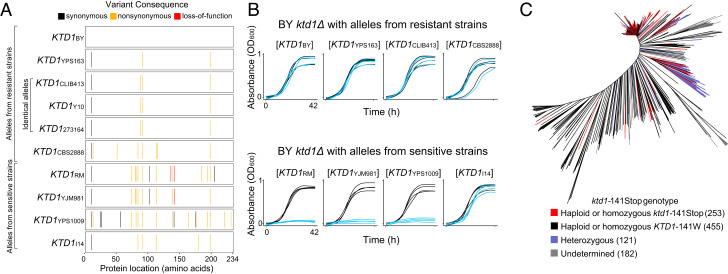
Analysis of *KTD1* alleles in the yeast population. (*A*) Genetic variants in each *KTD1* allele relative to the reference genome. YJM981 carries a nonsense variant that produces a premature termination codon at codon 141. RM carries a frameshift at codon 136 as well as the nonsense variant at codon 141, which is out of frame due to the frameshift (Fig. 2*B*, *inset, black dotted box*). (*B*) Growth curves of strains carrying the indicated *KTD1* allele on a centromeric plasmid in the BY *ktd1Δ* background; blue and black growth curves correspond to strains grown in minimal media with or without K28, respectively (*n* = 4). The subscript of the allele label indicates the strain in which that *KTD1* allele was found. ANOVA followed by Tukey’s HSD (*SI Appendix*, Fig. S7) identified the *KTD1*_RM_, *KTD1*_YJM981_, and *KTD1*_YPS1009_ alleles as significantly less resistant than the other five (*P* adjusted < 5 × 10^−14^ for those comparisons). (*C*) Distribution of *ktd1*-141Stop and wild-type *KTD1-*141W among 1,011 yeast isolates (28).

We next explored whether variation in *KTD1* affected K28 resistance across the remaining 14 isolates in our panel. We identified eight distinct *KTD1* alleles in our panel (Fig. 3*A*), four of which were found in K28-resistant strains, and another four in K28-sensitive strains. We first tested whether the *KTD1* alleles from resistant strains conferred toxin resistance upon the sensitive BY *ktd1Δ* strain, and found that all of them did (Fig. 3*B*). Next, we tested the *KTD1* alleles from sensitive strains. Expression of *KTD1* alleles from RM, YJM981, and YPS1009 did not protect against K28 toxin. *KTD1*_YJM981_ is disrupted by a premature termination codon at codon 141; this premature termination codon is quite common in the population, present in 37% of 1,011 *S. cerevisiae* strains sequenced in a recent population survey (28) (Fig. 3*C*). In contrast, the *KTD1*_YPS1009_ allele was intact, indicating that it was likely nonprotective due to its nonsynonymous or promoter variants. Interestingly, the *KTD1* allele from the sensitive I14 strain conferred K28 resistance. We inferred that strain I14 likely carries a sensitizing genetic variant elsewhere that overcomes *KTD1*_I14._ Analysis of the I14 genome revealed a frameshift mutation in *VAM7* (Dataset S1), whose deletion in the BY background causes hypersensitivity to K28 (19). Our results indicate that variation in *KTD1* function is a major determinant of K28 resistance across the yeast population. However, we note that the sensitivity of I14 as well as resistance of five strains (PW5, YJM454, YJM145, CLIB219, and YJM978) that lack *KTD1* (Figs. 1*B* and 3*B*) highlight additional genetic complexity in K28 resistance.

### Ktd1p’s Site of Action in the Cell.

**Fig. 4.**
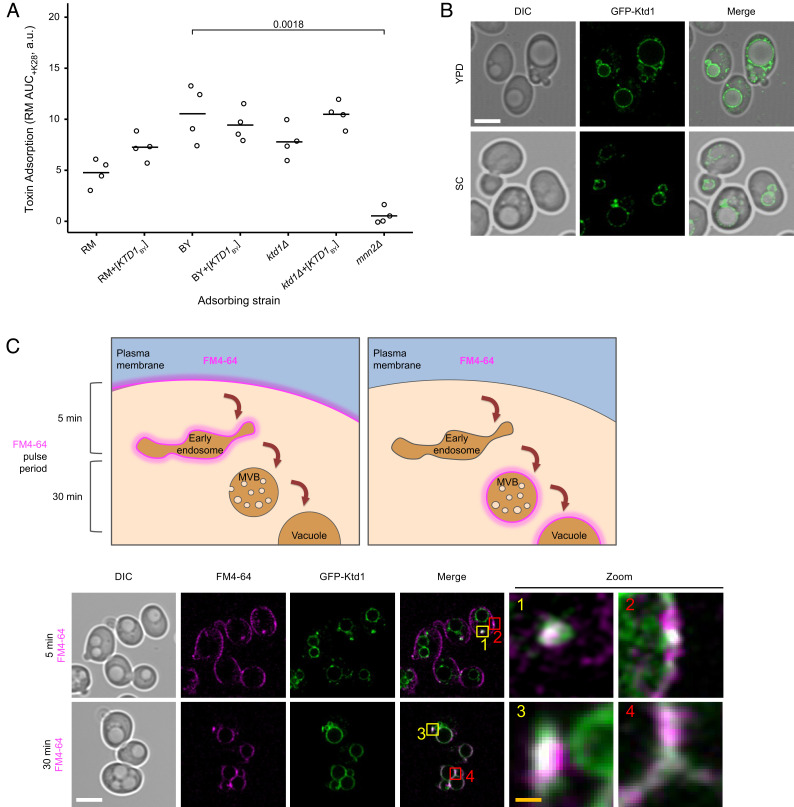
Analysis of Ktd1p’s site of action in the cell. (*A*) Ktd1p does not reduce adsorption of K28 to the cell surface. Media containing K28 was applied to cells carrying either *KTD1*_BY_ or empty vector in various genetic backgrounds (RM, BY, or BY *ktd1Δ* or *mnn2Δ*) (*n* = 4 biological replicates). After 15 min, the media was filter-sterilized to remove the cells and any K28 adsorbed onto their surfaces. The remaining, nonadsorbed K28 was quantified by its inhibition of growth of the K28-sensitive RM strain, measured as AUC_+K28_ from the growth curves in *SI Appendix*, Fig. S8. Higher values indicate more toxin was adsorbed by the strain. Statistical analysis was performed using Welch’s two-sample one-tailed *t* test, with the alternative hypothesis that *KTD1*_BY_ or *mnn2Δ* leads to decreased toxin adsorption. No significant decrease in K28 toxin adsorption due to *KTD1*_BY_ expression from a centromeric plasmid was found in any of RM, BY, or BY *ktd1Δ*, or for RM compared to BY, whereas BY *mnn2Δ* had significantly lower toxin adsorption than BY WT (*P* = 0.0018), as previously shown (19). (*B*) Confocal microscopy of wild-type BY cells expressing GFP-Ktd1p using an Airyscan2 detector. Cells were grown to log phase in rich (YPD) or SC media prior to imaging. (*C*) Colocalization of Ktd1p with components of the endolysosomal system labeled with the styryl dye FM4-64. (*Upper*) Schematic showing the pulse chase strategy to label early (5 min) and late (30 min) endocytic structures with the lipid dye FM4-64. (*Lower*) Cells expressing GFP-Ktd1p were grown to mid-log phase before labeling with 0.5 µM FM4-64 in YPD media for 10 min. Labeled cells were washed and grown for indicated times prior to imaging, with zoomed insets highlighting examples of Ktd1p colocalization with 1) endosomes, 2) cell periphery, 3) MVBs, and 4) the vacuolar membrane. White scale bar, 5 µm; orange scale bar, 1 µm.

We examined how Ktd1p protects against K28 lethality. K28 cytotoxicity first requires K28 to bind to the cell wall (20), so we tested whether Ktd1p counters K28 at this step or after. Using a toxin adsorption assay (19, 33), we assayed the ability of K28 to bind to the surface of strains with or without functional *KTD1*. We found that expression of *KTD1* did not reduce K28 binding (Fig. 4*A*) in either of the sensitive BY *ktd1Δ* or RM backgrounds, despite conferring K28 resistance to both (Fig. 2 *D* and *E*). Additionally, RM did not exhibit higher K28 binding than toxin-resistant BY. As a control, we recapitulated that deletion of the mannosyltransferase *MNN2* prevents K28 binding (19), consistent with the requirement of mannosylation of cell surface proteins for K28 binding. We concluded that *KTD1* functions after the initial binding of K28 to the cell surface, either by blocking uptake or by acting once the toxin is inside the cell.

To further pinpoint where Ktd1p might act to block K28 toxicity, we determined the intracellular localization of Ktd1p. Previous studies could not determine the localization of tagged Ktd1p expressed from its endogenous promoter (34, 35). Similarly, even with super-resolution microscopy, we did not observe any signal from endogenously expressed Ktd1p fused to either GFP or mCherry (*SI Appendix*, Fig. S9*A*). Instead, we used the *NOP1* overexpression promoter. GFP-Ktd1p expressed from the *NOP1* promoter was functional for K28 protection, as determined with a K28 sensitivity halo assay (*SI Appendix*, Fig. S9*B*). Visualizing GFP-Ktd1p showed it localized mainly to the vacuolar membrane (Fig. 4*B*). Colocalization studies of GFP-Ktd1p and the endolysosomal system, using compartment markers (*SI Appendix*, Fig. S10) and pulse labeling with the styryl dye FM4-64 (Fig. 4*C*), also indicated that Ktd1p is predominantly localized to the vacuolar membrane. Some Ktd1p signal was localized to the *trans*-Golgi network and endosomes/multivesicular bodies (MVBs). We also observed a small amount of peripheral signal that was distinct from eisosomes but overlapped with both the plasma membrane and the cortical ER. Overall, the results of these localization studies suggest that Ktd1p acts against K28 during its trafficking after endocytosis.

### Mapping of KTD1 Domains Critical for K28 Protection.

**Fig. 5.**
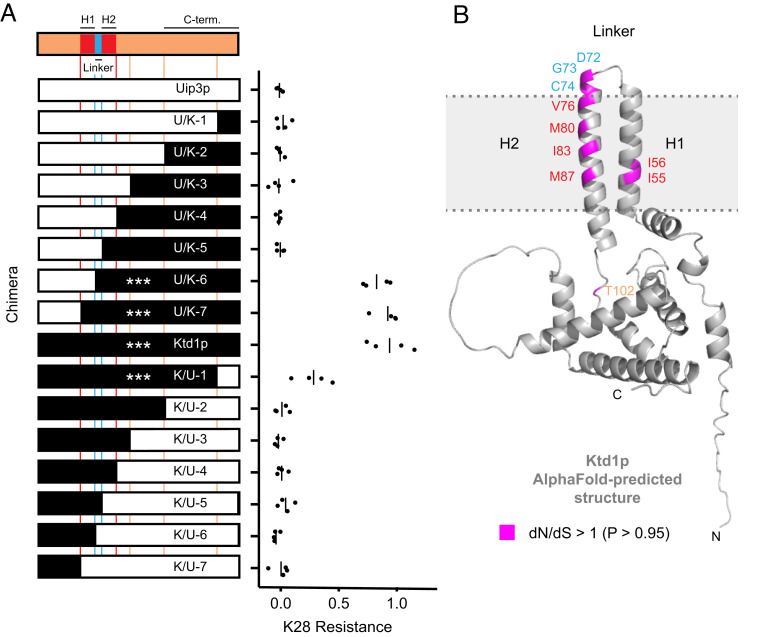
Ktd1p protein analysis. (*A*) K28 resistance phenotypes of chimeras between Ktd1p and Uip3p expressed on a centromeric plasmid from the *KTD1* promoter in the BY *ktd1Δ* background. White segments denote regions of chimeras derived from the nonprotective Uip3p, and black segments denote regions derived from the protective Ktd1p. Four biological replicates are shown for each chimera’s K28 resistance phenotype, measured as AUC_+K28_ / AUC_−K28_ from the growth curves in *SI Appendix*, Fig. S11, with vertical bars denoting the sample mean. ***P adjusted < 0.001 in comparison to Uip3p (ANOVA followed by Tukey’s HSD). (*B*) Sites of positive selection in Ktd1p. Sites having a greater than 0.95 probability of dN/dS above 1 are highlighted in magenta. The protein structure of Ktd1p was predicted by AlphaFold (36) (predicted aligned error shown in *SI Appendix*, Fig. S14). The colors of the amino acid labels correspond to the coloring of the topological domains of Ktd1p in part A, as defined by Poirey et al. (30). The transmembrane helices are labeled H1 and H2.

*KTD1* is a member of the enigmatic DUP240 gene family. None of the 10 DUP240 genes in the reference genome have a clearly ascribed function (*SI Appendix*, Table S3), and simultaneous deletion of all DUP240 genes does not affect yeast growth (30). Deleting *KTD1* severely abrogated K28 resistance in BY despite the presence of nine other DUP240 genes, suggesting that the other DUP240 genes in BY do not confer K28 resistance. Indeed, we found no contribution to K28 defense from Uip3p, the most similar DUP240 family member to Ktd1p (*SI Appendix*, Fig. S11): Deletion of *UIP3* did not reduce K28 resistance (*SI Appendix*, Fig. S5), and expression of *UIP3* did not increase resistance in a *ktd1Δ* background (Fig. 5*A*).

To identify the protein domains responsible for K28 protection, we constructed chimeras between Ktd1p and Uip3p (Fig. 5*A*). DUP240 proteins have two predicted transmembrane domains separated by a short inter-helix linker, as well as three conserved domains (30). We made chimeras that transition between Ktd1p and Uip3p at the boundaries of the transmembrane helices or the conserved domains (*SI Appendix*, Fig. S11*A*) and tested each chimera’s ability to protect against K28 in a *ktd1Δ* background. Our experiments identified two regions of Ktd1p important for protection: the inter-helix linker and the C terminus (Fig. 5*A*). When the 88 C-terminal amino acids of Ktd1p were replaced with the corresponding region of Uip3p (chimera K/U-2), K28 resistance was fully abrogated. In addition, chimeras U/K-5 and U/K-6, which differed only in the identity of their inter-helix linker, showed very different K28 protection ability (Fig. 5*A*), indicating the inter-helix linker is critical for K28 protection. The inter-helix linker region was of particular interest, as it is the only part of Ktd1p predicted to reside on the noncytoplasmic face of the membrane (37) and thus could affect K28 trafficking to the cytoplasm (20).

### Rapid Evolution in the DUP240 Gene Family.

Gene families involved in host defense often experience evolutionary pressure to change (38), which can lead to their expansion and contraction or elevated nonsynonymous mutation rate relative to synonymous mutation rate (dN/dS). We examined whether such evolutionary signatures were present in the DUP240 gene family*.* DUP240 genes recently arose in the *Saccharomycetaceae* family (*SI Appendix*, Fig. S12) (31). Within *S. cerevisiae*, DUP240 gene content varies significantly between strains in terms of gene identity, copy number, and genomic location (Dataset S2) (31). In addition to the 10 DUP240 genes annotated in the reference *S. cerevisiae* genome, we identified 16 further distinct DUP240 gene sequences from the strains in our 16-isolate panel, with strains having between 6 and 14 DUP240 genes (Dataset S2*A*). In addition to the previously identified DUP240 loci on chromosomes 1, 3, 7, and 8, we found a novel DUP240 locus on chromosome 12 in the strain PW5 containing three DUP240 genes (Dataset S2*B*). Intriguingly, with the exception of *DFP4* (YHL044W), DUP240 genes are not found near telomeres, unlike most gene families of variable composition in *Saccharomyces* (39). However, most DUP240s are found near transposon sequences and tRNA genes (31), which are known to facilitate recombination (40). The pattern of DUP240 expansion and contraction suggests the DUP240 family could be experiencing rapid evolution.

To investigate whether DUP240s are evolving rapidly at the amino acid level, we generated models of dN/dS ratios per codon from 18 DUP240 homologs closely related to *KTD1* (41). Ten codons were identified as having dN/dS >1, indicating positive selection at those sites (*SI Appendix*, Table S4). The corresponding residues are concentrated in the region spanning the transmembrane helices and the linker (Fig. 5*B* and *SI Appendix*, Fig. S13): Three are in the linker itself and six are in the adjacent transmembrane helices. Combined, the positive selection near the DUP240 inter-helix linker and the linker’s role in determining K28 resistance (Fig. 5*A*) suggest that this inter-helix linker could be evolving rapidly to maintain protection against killer toxins.

## Discussion

Protein toxins are powerful weapons in inter- and intraspecies competition that can lead to polymorphic adaptations in the targeted populations (4–7, 9, 42). We discovered a polymorphic yeast gene, *KTD1*, that protects yeast against the killer toxin K28. We found several distinct alleles of *KTD1* in the yeast population, some carrying a highly prevalent premature stop codon. Yeast are found in diverse environments and geographical locations. In environments where K28 is potent, such as low pH (20) and where yeast are at high density (43), we expect *KTD1* function to be maintained, whereas other environments might favor the loss of its function. Alternatively, there may be alleles of K28 in the killer yeast population that successfully evade Ktd1p, in which case *KTD1* would be dispensable in yeast that primarily encounter those alleles. Distinct alleles of K28 have been described (21), but it is not known whether they vary functionally. More work is required to understand how variation in K28 and *KTD1* affects the dynamics of yeast populations in different environments.

Our analysis strongly suggests that the DUP240 gene family, which includes *KTD1*, is under positive selection, with rapidly evolving sites concentrated in the transmembrane domains and the linker between them. We found that the linker is critical for Ktd1p-mediated resistance to K28 in chimeras between Ktd1p and a nonprotective DUP240, Uip3p. Notably, based on the predicted membrane topology of Ktd1p, the protection-determining linker resides on the luminal face of the membrane, and thus would be the only part of Ktd1p in the same cellular compartment as K28 prior to K28 entering the cytoplasm. If the linker physically interacts with K28 during K28 sorting as part of *KTD1*’s protective function, this could explain the rapid evolution in the linker region.

After binding to the cell surface, K28 is endocytosed but then avoids being targeted for vacuolar degradation (20), instead traveling to the endoplasmic reticulum before ultimately entering the cytoplasm where it exerts its toxic effect. Ktd1p could provide resistance by facilitating vacuolar degradation of endocytosed K28, consistent with our finding that Ktd1p acts after K28 binds to the cell surface. Two pieces of evidence support this idea. First, other DUP240s have been implicated in protein trafficking between the endoplasmic reticulum and Golgi apparatus (44) and in recycling membrane proteins to the plasma membrane (45). Second, and most intriguingly, the closely related Cos/DUP380 proteins are known to mediate degradation of endocytosed membrane proteins by trapping them in late endosomes and packaging them into MVBs destined for degradation in the vacuole (46, 47). Perhaps Ktd1p uses a similar mechanism to trap K28 for vacuolar degradation, though we note that Ktd1p predominantly localized to the membrane of the vacuole, whereas the Cos proteins localize to the vacuolar lumen following their cargo sorting function. Ktd1p’s localization at vacuolar and endolysosomal membranes suggests it either traffics K28 to the vacuole or acts to maintain it there.

A key question is whether *KTD1* is a dedicated killer toxin defense factor, or a housekeeping gene not directly involved in toxin defense but whose loss allows greater K28 effectiveness, as is the case for K28-hypersensitive proteasomal mutants (19). We favor the former possibility, as no previous function has been ascribed to *KTD1*, and it can be simultaneously deleted with all the other DUP240 gene family members without an apparent phenotype (30). Though we see Ktd1p localize to various structures in the endolysosomal system, we do not observe defects in cargo sorting for any post-Golgi membrane trafficking pathway when *KTD1* is deleted (*SI Appendix*, Fig. S15). Additionally, its evolutionary history, including its recent birth, sporadic absence in many strains, and the signature of rapid evolution, also points to its primary function being a defense function rather than a housekeeping function. Ultimately, distinguishing between these possibilities will require elucidating Ktd1p’s mechanism of action and a full understanding of K28’s mechanism of intoxication.

Our results indicate that additional strategies for K28 resistance exist in the yeast population. In addition to Ktd1p, there are a few known ways for yeast to be immune to K28. Killer yeast themselves are immune to the toxins they secrete. For K28, this involves an elegant mechanism in which K28 is initially expressed as a nontoxic precursor that is subsequently processed to the mature toxic form. The precursor can oligomerize with the mature toxin in the cytosol, leading to its degradation in the proteasome (25). In theory, this immunity mechanism could be adopted by nonkiller yeast if they acquired a gene encoding a nontoxic but protective K28 precursor, for instance, with a loss-of-function mutation in the cytotoxic domain. However, we have not found K28 in the nuclear genomes of any of our yeast strains. Perhaps some feature of the K28 gene renders it difficult to express from the nuclear genome, as has been seen for some fungal toxin genes with high A/T content (48). Or perhaps there is a higher cost to precursor-mediated immunity than *KTD1-*mediated immunity, such as if the K28 precursor retains some toxicity, or if it requires a stoichiometric excess to confer immunity (25). Many loss-of-function mutations are also known to confer K28 resistance, such as mutations in core pathways of cell wall biogenesis and endocytosis; a screen of the BY knockout collection identified 365 deletion or temperature-sensitive mutants that affected K28 sensitivity, including 176 resistant mutants (19). We scanned the sequenced genomes of our 16 strains for naturally occurring loss-of-function mutations in these genes, and identified that the genes *ICS3*, *SUL1*, and *HOC1* were truncated in some strains (Dataset S1). Future QTL mapping could quantitatively determine the degree to which these, and other, genetic variants contribute to the population-wide variation in K28 sensitivity. The most interesting strains for QTL mapping would include strains we identified that lack *KTD1* and M28 and yet are resistant to K28 toxin (Figs. 1 and 3 and *SI Appendix*, Figs. S2 and S3). In addition, just as there is natural variation in resistance to K28, the yeast population also varies in sensitivity and resistance across the range of known killer toxins (22). Overall, our findings have scratched the surface of natural adaptations in yeast that protect against killer toxins.

## Ideas and Speculation

The genes in the yeast genome are generally very well characterized, especially at the level of gene families. Here, we describe a function for *KTD1*, a gene in the DUP240 gene family, one of the few remaining large uncharacterized gene families in yeast. Our identification of *KTD1* as a killer toxin defense factor has demonstrated a protective role for a DUP240 gene against a killer toxin, which is among the few strong phenotypes that has been ascribed to any DUP240 family member. The evidence of positive selection within the family raises an intriguing possibility: Is the DUP240 gene family an anti-killer toxin innate immunity system? Perhaps the other genes in the family provide protection against other alleles of K28, or alternatively, given the diversity of killer toxins in yeast, they could even protect against entirely different killer toxins. If so, this could explain the high turnover and rapid evolution in DUP240 genes. When a novel allele of a killer toxin appears, duplication and rapid diversification of a DUP240 gene could protect against both the new and old versions of the toxin. Iterative duplication and diversification, possibly aided by proximity to transposons and tRNA genes (31, 40), would quickly generate a novel gene family experiencing positive selection at key protective sites. At the same time, the gene family could contract in yeast that stop encountering particular toxins. This pattern of large host defense gene families with fluctuating size is seen across biology, such as mammalian KRAB zinc-finger proteins that defend against transposons (49), bacterial restriction–modification systems that protect against phage and plasmids (50), and plant NBS-LRR genes that defend against pathogens (51). Nonetheless, gene families in host defense are uncommon in yeast; the only one we are aware of is the MDR family (52), which encode multidrug-resistance transporters. If the true purpose of DUP240s is to protect against killer toxins, then they would be a rare case of a yeast host defense gene family, indicating that killer toxins have had a significant impact on yeast evolution.

## Materials and Methods

### Strains.

Strains, plasmids, and oligonucleotide sequences used in this study are listed in Dataset S3. All constructed plasmids were sequence-verified using Sanger sequencing (Eurofins Genomics, Louisville, KY, USA).

The 16-isolate panel of diverse *S. cerevisiae* strains, MSY24-MSY39, was composed of stable haploid strains made by Bloom et al. (24) and were gifts from Leonid Kruglyak, as well as MSY1 and MSY8 and the panel of BY × RM segregants (29). MS300c (MSY20) and 192.2d (MSY21) were gifts from David Drubin (19).

We generated a diploid K28-secreting strain for production of toxin-containing supernatant (described in the section “*Preparation of Toxic and Nontoxic Supernatants*” below). As this supernatant was to be applied to haploid cells of either a or alpha mating type, we designed our K28-secreting strain to be diploid so that it would not release mating pheromone into the media. A K28 hypersecretor, MS300c [MATalpha *leu2 ski2-2* {M28 infected}] (53), was mated to *ski2Δ* (MATa *his3Δ leu2Δ met15Δ ura3Δ ski2Δ*::KanMX) from the MATa knockout collection (Transomic, Huntsville, AL) to produce a hypersecretor diploid (*ski2Δ*/*ski2-2*) strain, MSY52, that is infected with the M28 virus and preserves the hypersecreting phenotype due to the absence of *SKI2* function.

We generated a virus-cured strain for production of nontoxic control media. Virus curing was performed by growth of MSY52 at high temperature (54). MSY52 was pregrown in liquid YPD media overnight at 37 °C (elevated) or 30 °C (control). Cultures were diluted in fresh YPD to a density of approximately 500 cells/200 μL, at which point 200 μL of cultures were pipetted onto YPD agar plates. After a 2-day incubation at respective temperatures (30 or 37 °C), colonies were replica-plated onto Methylene Blue Agar (MBA) plates (pH 4.7) seeded with lawns of the K28-hypersensitive 192.2d strain (MSY21). All 30 °C control colonies showed killer activity, indicated by the inability of 192.2d to grow in their vicinity. Several colonies from the 37 °C plate no longer exhibited killer phenotype, one of which was selected and named MSY53. The absence of M28 virus was confirmed by virus typing assay (see below).

*uip3Δ*, *ktd1Δ* (MSY123), and *mnn2Δ* were taken from the MATa knockout collection.

*NOP1pro-GFP-KTD1* (CMY2365) was taken from the SWAp-Tag collection (35). Strains for colocalization studies (CMY2377, 2379, and 2380) were generated by crossing CMY2365 to strains expressing mCherry-tagged proteins with known localization. The strains carrying *KTD1* expressed from its native promoter and fused with GFP or mCherry (CMY2362, 2363) were generated by C-terminal tagging (55). The *ktd1∆* strain carrying *STE3-*GFP-DUb (CMY2390) was generated by knocking *STE3*-GFP-DUb::*HIS3* into the *his3Δ* locus. All transformations used standard lithium acetate transformation procedures (56).

The remaining strains in Dataset S3*A* were generated by transforming *ktd1Δ*, *mnn2Δ*, RM (MSY8), or BY (MSY1) with the plasmids described in Dataset S3*B* using standard lithium acetate transformation procedures.

### Stock Solutions and Media.

Phosphate-citrate buffer (pH 4.7) was prepared by first dissolving 56.88 g K_2_HPO_4_ into 200 mL distilled water and subsequently titrating with approximately 30 g citric acid until pH 4.7 was reached. The solution was sterilized by autoclaving for 20 min. Crystals may form over long periods of storage, which can be redissolved by heating the buffer to 80 °C for 20 to 30 min, stirring, and cooling.

Specialized low-pH complete supplement mixture (CSM) minimal media for production of K28 killer toxin, CSM pH 4.7 + 0.05% gelatin, was prepared by adding premixed CSM powders (Sunrise Science, Knoxville, TN) and YNB (BD, Franklin Lakes, New Jersey, USA) according to manufacturer instructions, dextrose to 2% (MP Biomedicals, Irvine, CA), and adding back uracil and leucine to the concentrations specified in Dunham et al. (57). pH was adjusted with 112 mL of pH 4.7 phosphate-citrate buffer per 1 L media. For additional K28 toxin stability, media were supplemented with 0.05% type B gelatin (MilliporeSigma, Burlington, MA, USA, Cat. #G9391-100G) as per Woods and Bevan (58). The mixture was heat-stirred for 2 h to allow gelatin to dissolve. All CSM media were sterilized using 0.22 µm polyethersulfone membrane filters (MilliporeSigma).

For use with supernatants, as described below, we made concentrated replenishment minimal media, 5xCSM pH 4.7, either with or without histidine as appropriate for the experiment. These media were prepared by dissolving either 0.8375 g CSM-Leu-Ura or 0.8125 g CSM-His-Leu-Ura powders (Sunrise Biosciences) in 222 mL distilled H_2_O, along with 125 mg leucine, 25 mg uracil, 8.375 g YNB, and 25 g dextrose. pH was adjusted with 28 mL phosphate-citrate buffer (pH 4.7). The solutions were stirred for approximately 20 min to ensure full dissolution of uracil crystals and subsequently filter-sterilized.

Yeast peptone dextrose (YPD) media contained 1% yeast extract (Thermo Fisher Scientific, Waltham, MA, USA), 2% peptone (BD), and 2% dextrose, and was sterilized by autoclaving. When used for plates, the media additionally contained 2% agar. Synthetic complete (SC) media were made with 2% glucose, YNB, and complete amino acid and base supplements (Formedium, Norfolk, UK).

YPD-based MBA plates (pH 4.7) were prepared as described in Amberg et al. (59). Briefly, 1% yeast extract, 2% peptone, and 2% agar were dissolved in 415 mL distilled water and autoclaved for 20 min. Following autoclaving, media was supplemented with sterile solutions of 25 mL 40% glucose, 4.2 mL of 4 mg/mL methylene blue dye (MilliporeSigma), and 56 mL phosphate-citrate buffer of pH 4.7. Correct pH was confirmed using a pH probe on an aliquot after buffer addition.

### Preparation of Toxic and Nontoxic Supernatants.

Single colonies of MSY52 (diploid K28-secretor) or MSY53 (virus-free diploid) were separately inoculated into 200 mL of CSM pH 4.7 + 0.05% gelatin and incubated for 24 to 48 h at 23 °C, 70 RPM. These conditions were necessary for generating stable, high-activity K28 toxin. For experiments with strains carrying a *HIS3* plasmid, CSM-His was used instead of CSM for supernatant preparations. Cultures were filtered with 0.22 µm polyethersulfone membrane filters into prechilled glass bottles, producing the toxic (MSY52) and nontoxic (MSY53) supernatants.

### Liquid Growth Assay for K28 Toxin Resistance.

Single colonies of yeast strains were inoculated and pregrown in 100 μL CSM or CSM-His (pH 7.0) for 24 h in CORNING 96-well plates prior to the experiment. 1.5 μL of saturated yeast cultures were transferred into 160 μL of either the toxic or nontoxic supernatant, supplemented with 40 μL 5xCSM pH 4.7 (with or without histidine) to replenish spent nutrients, for a total volume of 201.5 μL in each well. Strains were randomly distributed across the plate to avoid confounding of strain identity with position on plate. The one exception is the experiment comparing toxin sensitivity of *uip3Δ* and *ktd1Δ* to WT (*SI Appendix*, Fig. S5), in which each strain occupied a specific row on the plate. Absorbance (OD_600_) of each well was measured on a SPECTROstar Omega microplate reader equipped with a plate stacker (BMG Labtech, Ortenberg, Germany) over a period of 2 to 3 d at ambient temperature (~25 C) with 30 s of shaking at 600 RPM prior to each plate reading. Reads were taken with variable frequency depending on the number of plates being read, with cycle time ranging from 8 to 104 min. For each strain in an individual well, the K28 toxin resistance phenotype was calculated, following blank correction, as the ratio of the area under the growth curve in toxic media (AUC_+K28_) to the area under the growth curve in nontoxic media (AUC_−K28_), i.e., AUC_+K28_/AUC_−K28_. Values close to 0 indicate low K28 toxin resistance, while values close to 1 correspond to high resistance.

### Killer Spotting Assay.

Four milliliters of YPD cultures of MSY21, MSY52-53, and MSY24-39 were grown overnight, with MSY39 (ade^−^) supplemented with 1% adenine. 2 × 10^7^ or 4 × 10^7^ cells of K28-hypersensitive MSY21 (192.2d) were uniformly spread on 10-cm MBA agar plates, with three replicate plates for each plating density. Cultures of MSY52 (K28-secreting control), MSY53 (virus-free control), and MSY24-39 (the 16-isolate panel) were pelleted at 3,000 rpm and resuspended in fresh YPD to make a ~50% v/v cell slurry, as previously described (19). One hundred and fifty microliters of these slurries were pipetted into wells of a 96-well plate and pinned with a VP407AH metal replicator (V&P Scientific, San Diego, CA) to transfer approximately 3 μL of slurry onto the previously MSY21-seeded MBA plates. Each spot of pinned cells was imaged through microscope oculars using the back camera of a Galaxy S10e mobile phone (Samsung, Seoul, South Korea) with Open Camera v1.49.1 software, which allowed for imaging of colonies at fixed exposure and white balance within an individual plate.

### Viral RNA Typing Assay.

To assay for presence of M28 virus in a given strain, reverse transcription PCR-based virus typing was performed as described by Chang et al. (21). Briefly, following isolation of total RNA with the Qiagen RNeasy Mini Kit (Qiagen, Valencia, CA, USA), complementary DNA (cDNA) was synthesized by incubating RNA for 2 h at 37 °C using the High-Capacity cDNA Reverse Transcription Kit (Applied Biosystems, Foster City, CA, USA). Afterwards, PCR with Kapa HiFi HotStart ReadyMix (Roche, Basel, Switzerland) was performed on the cDNA using M28 virus-specific primer pairs: oIA9/oIA10, oIA11/oIA12, and oIA13/oIA14 with expected fragment sizes of 253, 180, and 350 bp, respectively. These primers correspond exactly to M28-F1/R1, M28-F2/R2, and M28-F3/R3 primers used by Chang et al. The presence of M28 virus was characterized by strong PCR bands at expected amplicon lengths. A band of low intensity at 650 bp appeared in several experiments, which we deemed an artifact because its presence was irrespective of the particular PCR primer pair.

### QTL Mapping.

QTL mapping was performed on 960 haploid F1 segregants generated from a cross between BY and RM, which were a gift from Leonid Kruglyak (29). The segregants, arrayed in ten 96-well plates, were transferred in parallel with a disposable plastic pinner into 200 μL CSM pH 4.7 + 0.05% gelatin media either with K28 (10 plates) or without K28 (additional 10 plates), as described in the “Liquid Growth Assay for K28 Toxin Resistance” section above. Growth was measured in parallel over 63 h on a SPECTROstar Omega microplate reader equipped with a plate stacker. The K28 resistance of each segregant was quantified as the ratio of AUC_+K28_/AUC_−K28_, as described above. QTL mapping was performed by calculating the logarithm-of-odds (LOD) score for each of 28,220 biallelic markers, defined as

$$LOD=\frac{-\;N}{2\;ln\left(10\right)}ln\left(1-r^{2}\right),$$

where *N* = 912 is the number of segregants after filtering out ill-behaved segregants, and *r* is the Pearson correlation between the K28 resistance phenotype and the genotype of each segregant at that biallelic marker; see page 454 of Lynch and Walsh (60). Genotypes were determined by Bloom (29), and genotype data is available at https://github.com/gouliangke/Mutation-rate/. Ill-behaved segregants were identified post hoc on the basis of poor growth in media lacking K28 (34 strains), or lacking genotype information (14 additional strains). The genotypes are coded with −1 representing that the segregant had the BY allele and +1 signifying the RM allele, as determined by Bloom et al. (29). The significance threshold was determined by a permutation test: QTL mapping was done 1,000 times on permutations of the strain phenotypes relative to the strain genotypes, and the top LOD score was taken from each mapping. Among these LOD scores, the 950th highest value was taken as the 5% family-wise error rate (FWER) threshold for significance. Three QTLs were detected with peak LOD scores above this threshold. 95% confidence intervals for the location of the peak of these QTLs were determined by bootstrap with 1,000 samplings with replacement (61), taking the peak marker per QTL-containing chromosome from each bootstrap mapping, ordering them from left to right, and determining the confidence interval as the span between the 2.5th and 97.5th percentile bootstrap peaks.

### Cloning of KTD1 Alleles.

*KTD1* alleles tested in Figs. 2 *D* and *E* and Fig. 3*B* were amplified from genomic DNA using PfuUltraII Fusion HS DNA Polymerase (Agilent, Santa Clara, CA, USA) and primers listed in Dataset S3*C*. Genomic DNA was isolated using DNeasy Blood & Tissue Kit (Qiagen) and following Qiagen’s Supplementary Protocol “Purification of total DNA from yeast using the DNeasy Blood & Tissue Kit.” Primers used for amplification are listed in Dataset S3C (oIA44, 45, and 137), and amplify *KTD1* alleles along with their native promoters and terminators. Amplicons were Gibson assembled (New England Biolabs, Ipswich, MA, USA) with the centromeric plasmid pRS313 (MSp64) digested with XbaI-HF and XhoI-HF restriction enzymes (New England Biolabs) or with MSp101 (*KTD1*_BY_) digested with NotI-HF and ApaI-HF restriction enzymes (New England Biolabs).

### Toxin Adsorption.

To test the ability of K28 toxin to bind to the surface of yeast cells, we adapted the toxin-cell binding assay described in Carroll et al. and Breinig et al. (19, 33). Strains MSY94, 96 to 100, and 104 (*n* = 4 biological replicates) were grown to saturation for 48 h in 30 mL CSM-His (pH 7.0) at 30 °C, shaking at 200 RPM. In parallel, 200 mL of toxic and nontoxic supernatants were prepared as described earlier, with 24 h incubation time. The strain MSY26 (RM) was grown for 24 h in YPD. 10^9^ cells (50 OD_600_ units) of each experimental strain were pelleted at 3,000 RPM. Supernatant was mostly aspirated, leaving approximately 0.5 to 0.7 mL media in the tube. The pellet was resuspended in the remaining supernatant and placed on ice.

Five milliliters of toxic supernatant was added to each cell suspension and incubated for 15 min on a nutator at approximately 1 RPM. As controls, we also incubated cell-free toxic and nontoxic supernatants. Suspensions and cell-free controls were then filtered using syringes with 0.22-μm Nalgene syringe filters (Thermo Fisher Scientific), generating filtrates of dissolved toxin that had not adsorbed to cell surfaces. The toxin adsorption and filtration steps were done at 4 °C. The degree of toxin adsorption was quantified as the area under the growth curve (AUC_+K28_) of the K28-sensitive strain RM in 160 μL filtrate + 40 μL 5xCSM pH 4.7 for 48 h (*SI Appendix*, Fig. S8).

### Confocal Microscopy.

Cells were grown to mid-log phase and resuspended in fresh synthetic complete (SC) media prior to imaging experiments. Cells were loaded to a glass slide and imaging was performed using a Zeiss 980 laser-scanning confocal instrument equipped with an Airyscan2 detector and a 63× zoom Plan-Apochromat objective lens with 1.4 Numerical Aperture (Carl Zeiss AG, Oberkochen, Germany). YPD containing 1 μM FM4-64 (*N*-(3-Triethylammoniumpropyl)-4-(6-(4-(Diethylamino) Phenyl) Hexatrienyl) Pyridinium Dibromide) dye (Thermo Fisher Scientific) followed by washes in SC media was used to label the endolysosomal system, with chases performed in media lacking dye for either 5 or 30 min. SC media containing 50 µM CMAC (7-amino-4-chloromethylcoumarin) CellTracker Blue dye (Thermo Fisher Scientific) was used to label the vacuolar lumen. Blue, green, and red fluorescence was excited using solid-state lasers, using excitation wavelengths of 405 nm, 488 nm, and 561 nm, respectively, with emissions measured at wavelengths of 422/497 nm, 460/550 nm, and 570/620 nm, respectively.

### FM4-64 Recycling Assay.

Cells harvested from mid-log phase were concentrated 10-fold and resuspended in YPD containing 40 µM FM4-64 dye for 8 min at room temperature. Cells loaded with dye were washed in ice-cold minimal media three times for 5 min each, prior to washed pellets being resuspended in 3 mL room temperature minimal media for flow cytometry using a LSR Fortessa (BD). Cells flowed at a rate of approximately 1 million cells per minute at 600 V, and fluorescence was measured with 561 nm excitation and collected via 710 nm / 50 laser filter. Data were analyzed and efflux graphs plotted using FCS Express version 7 (de Novo Software, Pasadena, CA, USA).

### Chimeragenesis.

All chimeras between *KTD1* and *UIP3*, as well as *KTD1* and *UIP3* themselves, were expressed from the *KTD1* promoter (nucleotide position −311 to −1 relative to the *KTD1* start codon) and terminator (position +1 to +230 relative to the *KTD1* open reading frame (ORF)). Fragments were assembled via Gibson assembly onto centromeric plasmids. “Insert” PCR amplicons were generated using the high-fidelity PfuUltraII Fusion HS DNA Polymerase, while the longer “plasmid” PCR amplicons were generated using Herculase II Fusion DNA Polymerase (Agilent). Primer sequences used for chimeragenesis are listed in Dataset S3*C*.

To construct chimeras, first, we used the *KTD1* plasmid, MSp101, to construct the *UIP3* plasmid, MSp160: we Gibson assembled the *UIP3* coding sequence amplified from BY genomic DNA with an amplicon of plasmid MSp101 to replace the *KTD1* coding sequence with *UIP3*. To generate chimeras, we amplified a plasmid fragment from MSp101 that contained a segment of *KTD1* along with the plasmid backbone. We amplified the corresponding insert fragment from MSp160 which contained the desired *UIP3* segment. The chimera-specific primers used to produce each plasmid-insert pair of PCR amplicons gave them approximately 20 bp of homology at each end. These fragments were Gibson assembled. Chimera transition points were selected to according to the conservation- and topology-based domains of DUP240 proteins defined by Poirey et al. (30). Each plasmid was transformed into BY *ktd1Δ* and tested as described in the section “Liquid Growth Assay for K28 Toxin Resistance.”

### Identification of DUP240 Homologs.

To find all DUP240 genes in the 16-isolate panel, we examined genome sequences generated for each of the strains using long-read sequencing, in the form of PacBio (Pacific Biosciences, Menlo Park, CA, USA) circular consensus sequence reads. The generation and analysis of these genome assemblies are described in more detail in Weller et al. (62).

The 10 DUP240 genes from the reference *S. cerevisiae* genome were used as BLASTn (BLAST+ v2.10.0) queries against each of the assembled genomes with –*word_size 7*, but otherwise default parameters. Newly uncovered DUP240 homologs were recursively added to the search query over three iterations until no new homologs were found, leading to 16 new DUP240 homologs (*DFP11-DFP26*)*.* New homologs were defined primarily by having less than 96% nucleotide identity to all previous DUP240 homologs across at least 650 nucleotides. We additionally called some BLAST hits as alleles of existing DUP240s rather than new homologs despite having less than 96% identity if they met the following criteria:

*1. Overall nucleotide identity is between 70% and 96%*
*2. Same length*
*3. Large region (>50%) of high nucleotide identity (>99%)*
*4. Same genomic context*

If all of the above are satisfied, then the query and the BLAST hit are allelic.

To determine which BLAST hits were complete genes, sequences were analyzed using the *predORF* function in systemPipeR package (63). In the process of adding new DUP240 homologs, when multiple alleles of a novel homolog were found, we designated one allele as the “reference” allele based on ORF length and whether the strain was resistant to K28. All the code pertinent to this analysis is available on Zenodo at https://zenodo.org/record/7591105 (64).

As an orthogonal search strategy, since most DUP240 genes are found in arrays, we examined all predicted ORFs in each strain’s genome on chromosome I between *CDC15* and *YAT1* and chromosome VII between *ERV14* and *TYW3*, which flank the two DUP240 arrays in the reference genome. This did not uncover any additional DUP240 homologs.

Sequences of nonreference DUP240 homologs are available through the Zenodo archive (64) and have been submitted to the Saccharomyces Genome Database (SGD), with names *DFP11-26* (DUP240 family protein), and to GenBank, with accession numbers OQ364005-OQ364020 (65).

### Analysis of Positive Selection.

A codon-based alignment of the DUP240 genes was generated using PRANK (v.170427) (66) with the gaprate parameter set to 0.000001 and a gap extension penalty of 0.001. As the codeML module of the Phylogenetic Analysis by Maximum Likelihood (PAML) program package does not estimate dN/dS values for codons aligned to gaps, DUP240 genes that created lots of gaps in the alignment were removed, resulting in a final alignment of 18 DUP240 homologs (*SI Appendix*, Fig. S13). The analysis of BLASTn results showed that some of these homologs are recombinants, whose inferred phylogeny may be incorrect. We performed recombination analysis using Genetic Algorithm for Recombination Detection (GARD) (67) to predict recombination breakpoints. For breakpoints that occurred in the middle of a codon, we rounded to the nearest codon boundary. Seven breakpoints were identified, which we used to break the alignment into eight segments for further analysis.

Phylogenetic trees were generated on each segment with FastTree (2.1) (68) with *-gtr -gamma* options. We then used the codeml module in PAML (v 4.9j) to generate models M7 (no positive selection) and M8 (with positive selection) on each segment with the corresponding segment-specific alignment and phylogenetic tree. The two models were compared by computing the likelihood-ratio test statistic,

$$\lambda_{\mathrm{LR}}=2\left[ln\left(L_{M8}\right)-ln\left(L_{M7}\right)\right],$$

which is chi-squared–distributed with two degrees of freedom. The results are summarized in *SI Appendix*, Table S4.

Additionally, we investigated the possibility of recombination sites missed by GARD, which could potentially affect dN/dS estimation. We therefore manually inspected the alignment of the two segments that had positive selection in order to find homoplasies that could have been caused by recombination. Our analysis found two such potential homoplasies. We reran codeml with a modified sequence of *DFP21* in the window of 214 to 221 nt that matched *DFP23*, which removed the potential recombination. The results were not substantially changed: the M7 model for that segment is still rejected in favor of the M8 model, with a *P*-value of 2.618 × 10^−8^ and a dN/dS value of 1.9403 for the segment.

### Software, Statistical Analysis, Data Deposition, and Data Visualization.

Statistical analysis and data visualization were performed using custom R scripts, available at the Zenodo archive (64) and at https://github.com/ilya-andreev2/killer-virus. For experiments with comparisons among many strains, statistical analysis was performed using one-way ANOVA followed by Tukey’s post hoc HSD test. For experiments comparing specific pairs of strains, statistical analysis was performed with Welch’s two-sample *t* tests. The number of biological replicates used in each experiment (three to five replicates) was chosen to provide robust statistical power given the expected effect sizes and accounting for the statistical methods used.

Growth curves were generated using the *geom_smooth* function in the R package ggplot2 (version 3.3.5) (69). Area under the curve (AUC) was calculated using the *AUC* function (trapezoid method) from the R package DescTools v.0.99.42. Neighbor-joining phylogenetic trees of 1,011 sequenced yeast strains (28) were generated using the function *bionj* from the R package ape (version 5.3) (70). The maximum edge length was set to 0.7 to truncate a highly diverged clade of yeast strains. Jittered one-dimensional scatter plots were generated using the *ggboxplot* function in R package ggpubr. Protein sequence alignments were generated in Clustal Omega (71). The structure of Ktd1p was predicted using AlphaFold (version 2.0.0) (36) and visualized in PyMOL (version 2.3.0 Open-Source), with the position of the transmembrane helices shown according to Poirey et al. (30).

Nonreference DUP240 gene sequences have been deposited in GenBank with accession numbers OQ364005-OQ364020 (65). They are present on SGD named *DFP11* through *DFP26* (*D*UP240 *f*amily *p*rotein), with systematic names YSC0056 through YSC0071. Unnamed DUP240 family genes in the *S. cerevisiae* reference genome (YAR023C, YAR029W, YCR007C, and YHL044W) have also been given *DFP* names on SGD (*DFP1* through *DFP4*, respectively).

## Supplementary Material

Appendix 01 (PDF)

Dataset S01 (XLSX)

Dataset S02 (XLSX)

Dataset S03 (XLSX)

## Data Availability

All data and code has been archived in a permanent, independent repository located at https://zenodo.org/record/7591105 (64).  Sequences of *DFP11-26* have been deposited in GenBank with accession numbers OQ364005–OQ364020 (https://www.ncbi.nlm.nih.gov/nuccore/) (65).
