# Supplementary material for: Discovery of a rapidly evolving yeast defense factor, KTD1, against the secreted killer toxin K28
Source: Proc Natl Acad Sci U S A. 2023 Feb 17;120(8):e2217194120. doi: 10.1073/pnas.2217194120 (PMC9974470; doi:10.1073/pnas.2217194120)
Supplement: Supplementary file 1 — Appendix 01 (PDF) [file pnas.2217194120.sapp.pdf]

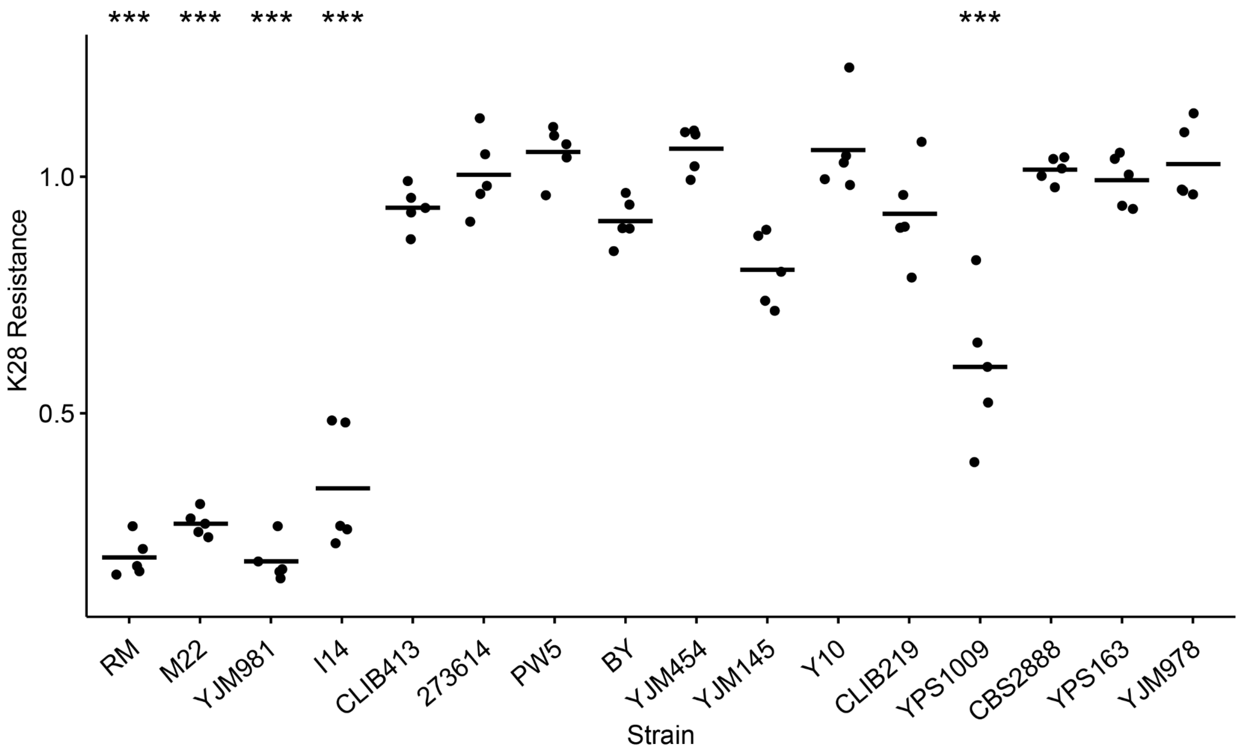

Supplementary Figure 1. Statistical analysis of K28 resistance of 16 diverse S. cerevisiae strains.

From the growth curves in Fig. 1B, K28 resistance was quantified as AUC_{+K28} / AUC_{-K28} with *n* = 5 biological replicates per strain. One-way ANOVA indicated a statistically significant effect of strain on K28 resistance (K28 resistance ~ strain). Tukey's post-hoc HSD test was performed to test for the difference in mean K28 resistance between all pairs of strains. Strains with significantly lower K28 resistance are marked with asterisks (***$P < 6.6\times10^{-6}$ in comparison to BY). Horizontal bars indicate the sample means.

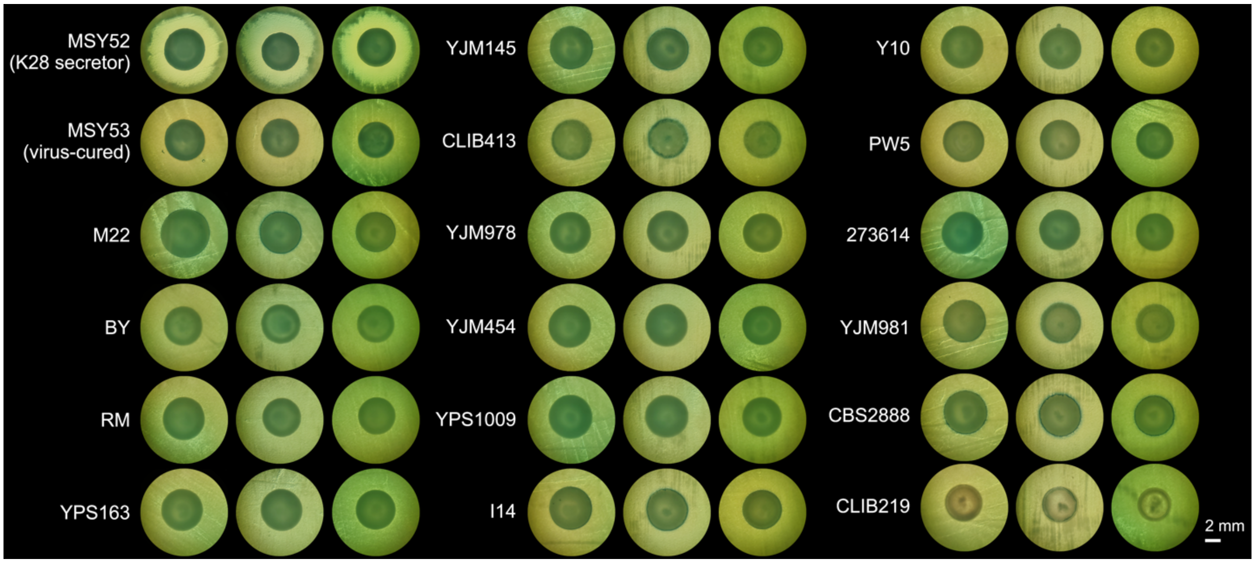

Supplementary Figure 2. Phenotypic Virus Typing – Halo Assay.
The 16-isolate panel was tested for production of K28 toxin by whether they killed the hypersensitive yeast strain 192.2d. Triplicate spots of each strain were grown on a lawn of 192.2d cells; strains producing K28 will generate a halo of cleared 192.2d cells (Carroll *et al.* 2009). Replicate 2 (middle column) is shown in Fig. 1C. MSY52, a diploid *ski2Δ* strain infected by M28, was spotted as a positive control; MSY53, a virus-cured derivative of MSY52, was spotted as a negative control.

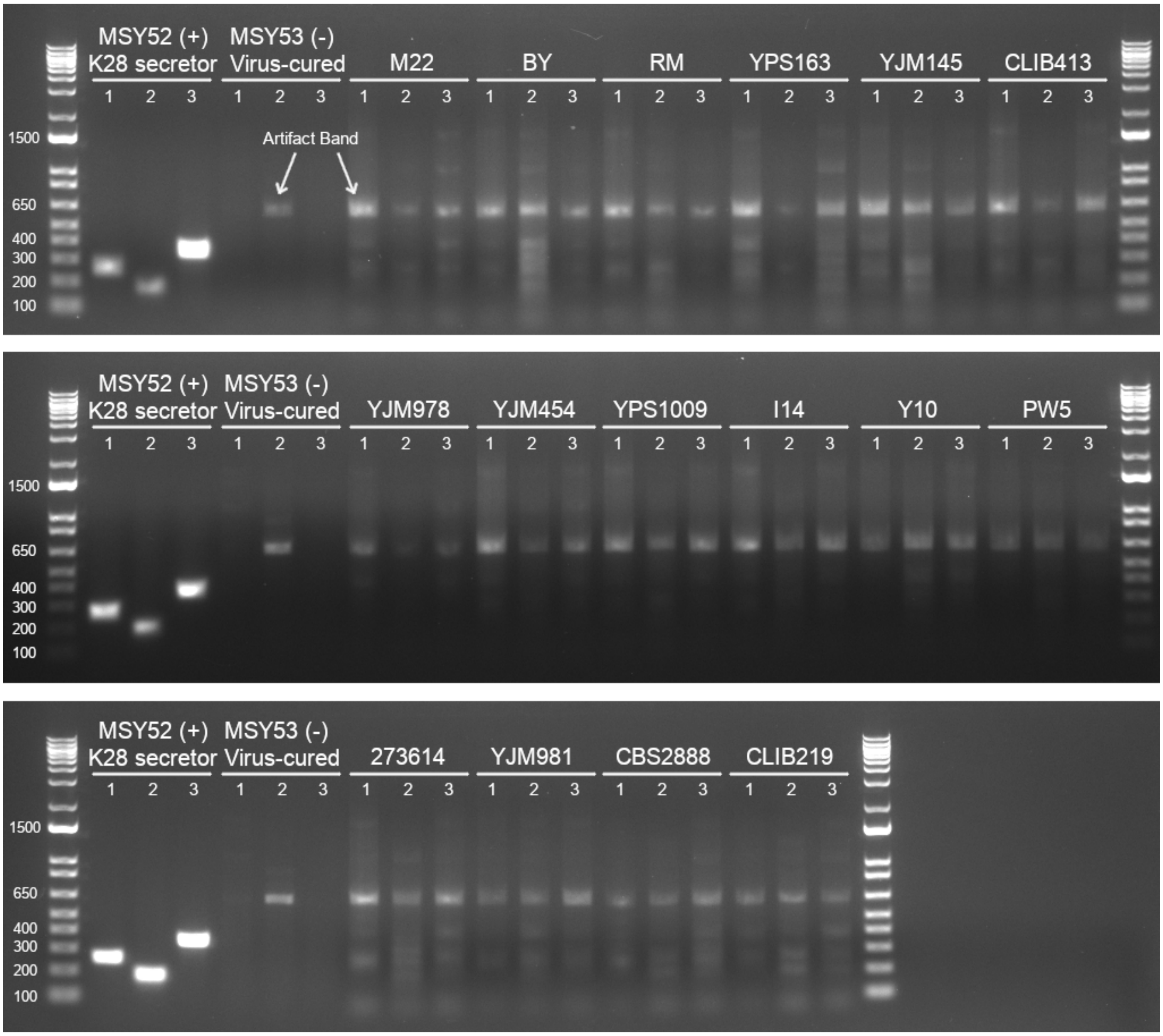

Supplementary Figure 3. RT-PCR-based Virus Typing.
PCRs designed to amplify segments of the M28 viral genome were performed on cDNA prepared from the 16-isolate panel and two control strains. Three distinct primer pairs were used for each strain, as described earlier (Chang *et al.* 2015), labelled '1,' '2,' and '3,' in the gel. Primer sequences are provided in Supplementary Dataset 3c (PCR 1: expected size of 253 bp; PCR 2: expected size of 180 bp; PCR 3: expected size of 350 bp). Ladder is 1kb Plus (Invitrogen). Shown gel is representative of n = 3 biological replicates, derived from three colonies of each strain.

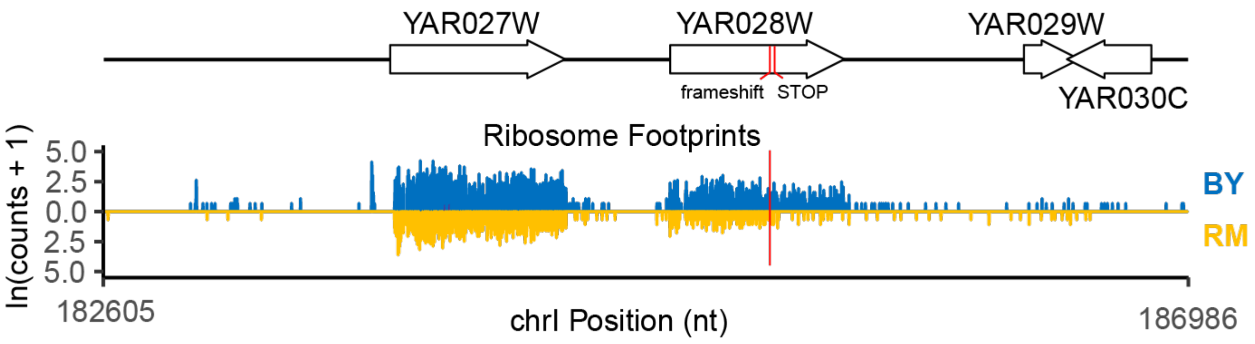

Supplementary Figure 4. Ribosome profiling of BY and RM strains near the YAR027W (*UIP3)* and YAR028W (*KTD1*) genes.

Log-transformed counts of ribosome footprints (Albert *et al.* 2014), are shown in a window of chromosome I encompassing *UIP3* (YAR027W) and *KTD1* (YAR028W). Footprints are plotted in blue for BY and gold for RM. In the gene diagram at top, the positions of deleterious mutations in RM are marked with vertical red lines, and in the ribosome footprint count data, the position of the frameshift mutation in RM is shown with a vertical red line. Count data are available at https://genome.ucsc.edu/s/falbert/FP_sacCer3

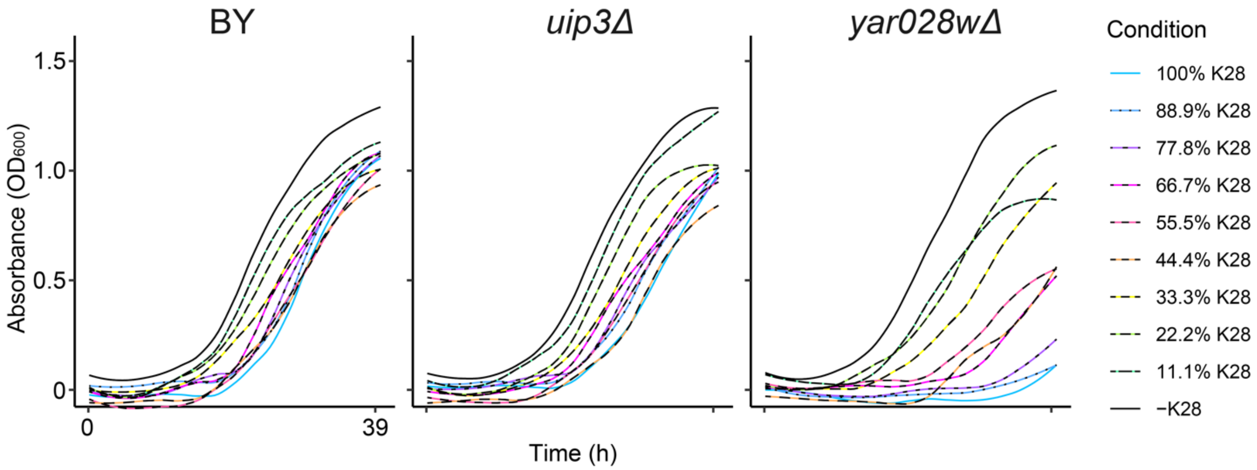

Supplementary Figure 5. Deletion of *UIP3* had no effect on K28 resistance.
BY, BY *uip3Δ*, and BY *yar028wΔ* (*ktd1Δ*) were grown in media containing different concentrations of K28, generated by mixing the supernatants of K28-producing (MSY52) and virus-free (MSY53) yeast strains. Legend indicates the percentage of toxic MSY52 supernatant (X%) diluted in non-toxic MSY53 supernatant (100% - X%).

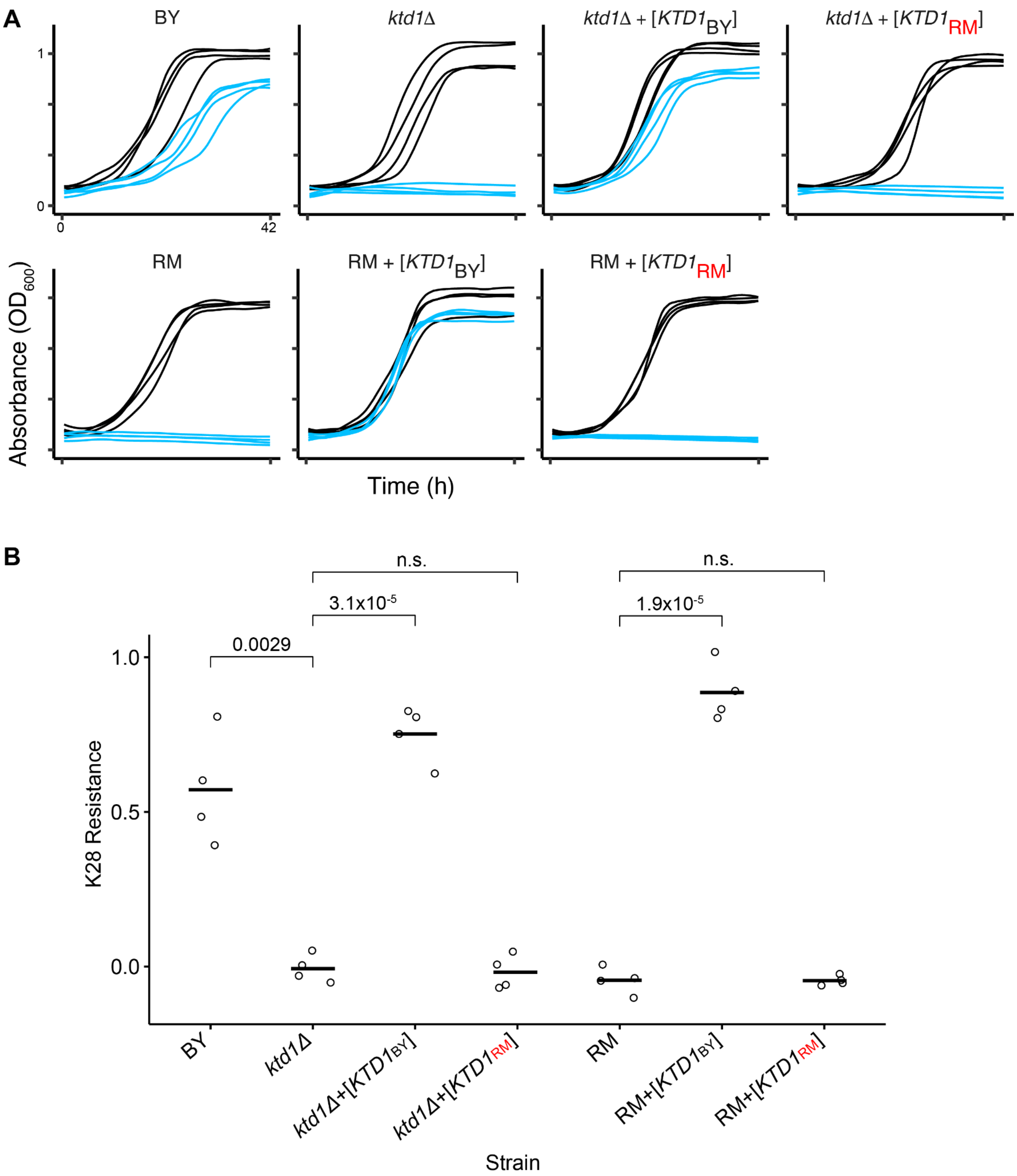

Supplementary Figure 6. Expression of the RM allele of *KTD1* did not protect against K28. **A,** Growth curves of BY, RM, and BY *ktd1Δ* are shown, expressing either the BY or RM allele of *KTD1*, or empty vector. Blue curves correspond to growth in media containing K28; black curves correspond to growth in media without K28, with n = 4 biological replicates per strain. A subset of these curves is also shown in Fig. 2 D and E. **B,** From the growth curves shown in part A, K28 resistance was quantified as AUC_{+K28} / AUC_{-K28}. Welch's two-sample *t*-tests were used to compare levels of K28 resistance between strains.

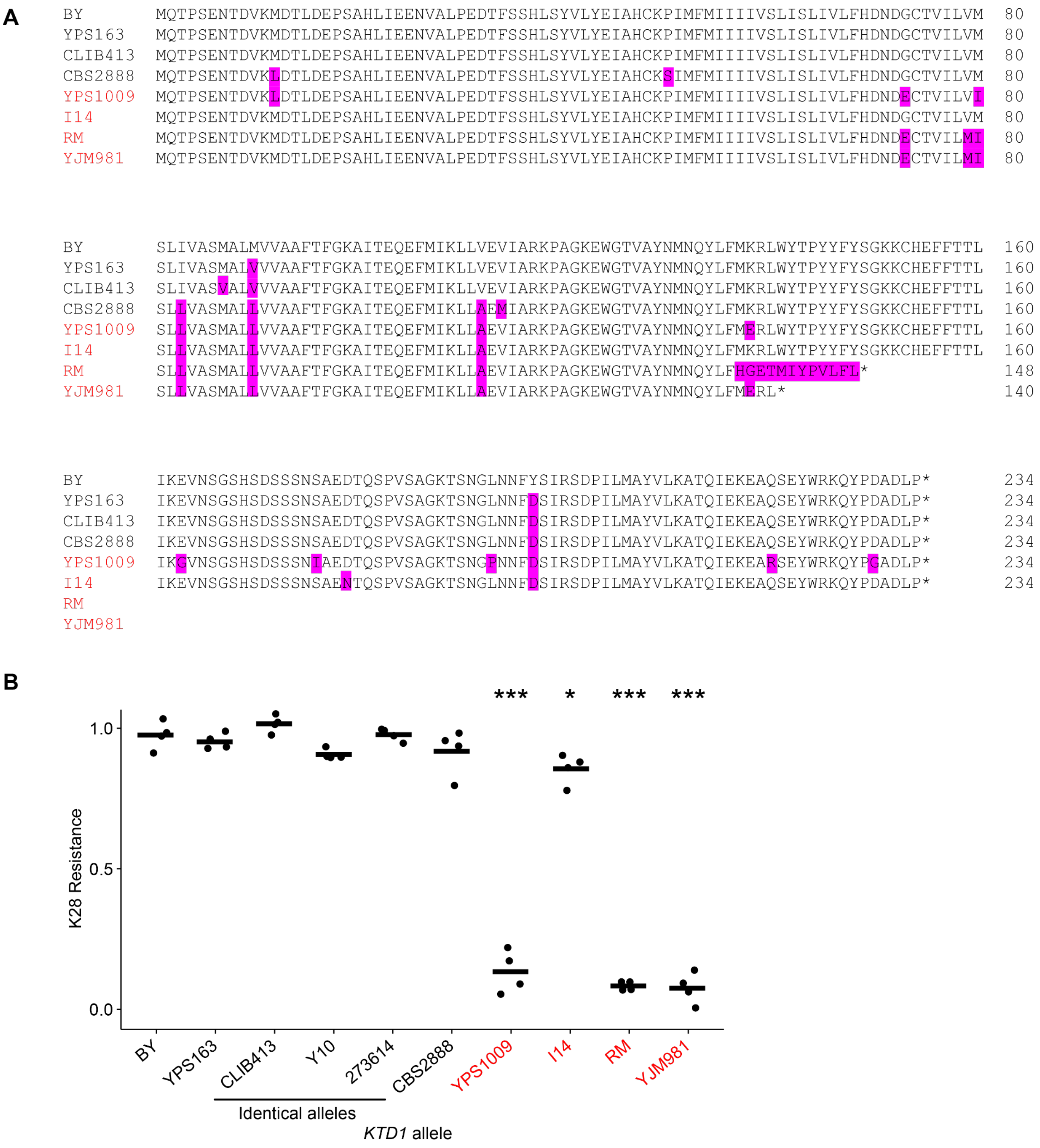

Supplementary Figure 7. *KTD1* alleles from the 16-isolate panel.
Strain names are colored according to K28 resistance as determined in Fig. 1B (black, resistant; red, sensitive). **A,** Amino acids that differ from the reference sequence (BY) are highlighted in magenta. Strains Y10 and 273614 had *KTD1* alleles identical to CLIB413 and are not shown here. **B,** From the growth curves shown in Fig. 3B of BY *ktd1Δ* expressing various *KTD1* alleles, K28 resistance was quantified as AUC_{+K28} / AUC_{-K28} with *n* = 4 biological replicates per strain. ANOVA followed by Tukey's HSD identified the six alleles from resistant strains and $KTD1_{I14}$

as conferring more resistance than $KTD1_{YPS1009}$, $KTD1_{RM}$, and $KTD1_{YJM981}$ (***$P < 0.001$ for all 21 pairwise comparisons). $KTD1_{I14}$ conferred less resistance than $KTD1_{BY}$ (*$P < 0.05$), $KTD1_{273614}$ ($P < 0.05$), and $KTD1_{CLIB413}$ ($P < 0.01$) at more moderate thresholds for statistical significance, but was not significantly different from the alleles from other resistant strains.

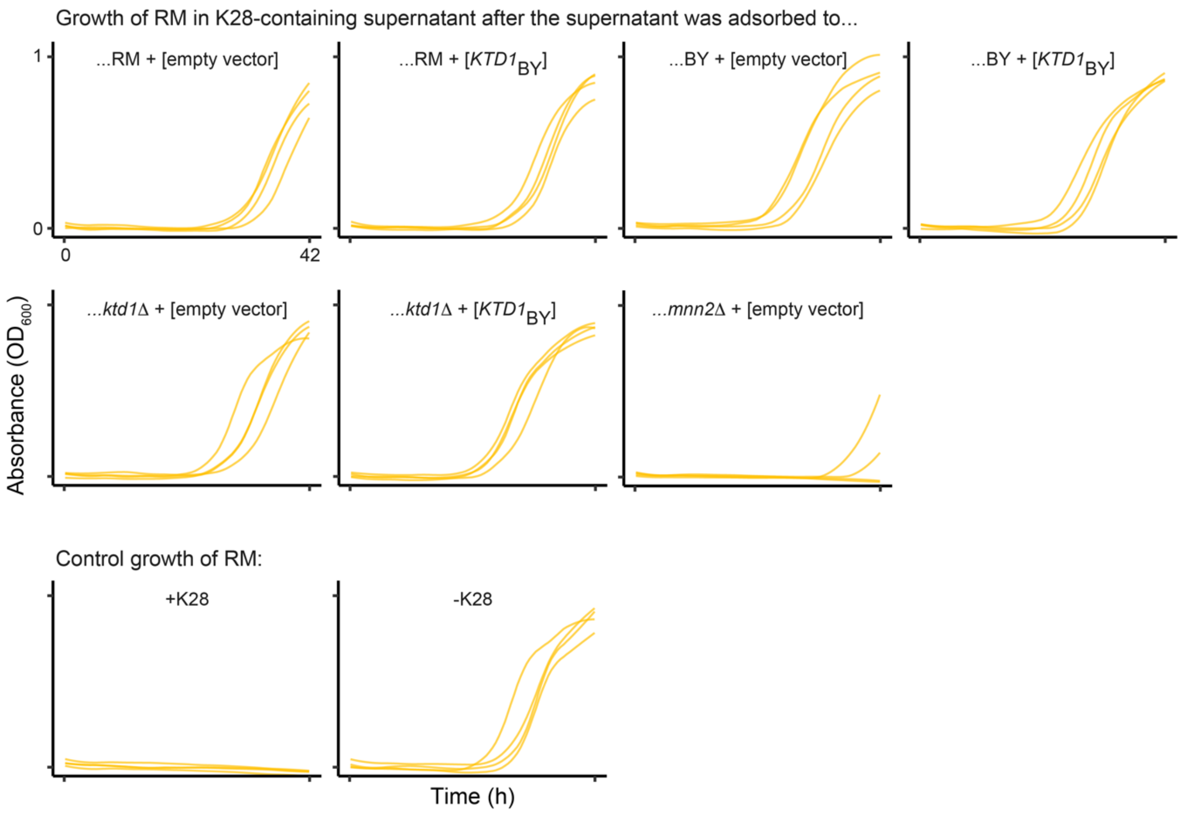

Supplementary Figure 8. Effect of Ktd1p on adsorption of K28 to the cell surface. Supernatant from the K28-secreting MSY52 strain was applied to cells of the genotypes shown (n = 4 biological replicates) for 15 minutes, after which the supernatant was filter-sterilized to remove the cells and any K28 adsorbed onto their surface. The growth curves show the ability of the K28-sensitive RM strain to grow in the given supernatant, which reflects the level of remaining K28 toxin following toxin adsorption. This growth ability was quantified as the area under curve (AUC), with quantification and statistical analysis shown in Fig. 4. As controls, the growth of RM was determined in supernatant of MSY52 (toxin secretor) and MSY53 (toxin-free) without an adsorption step.

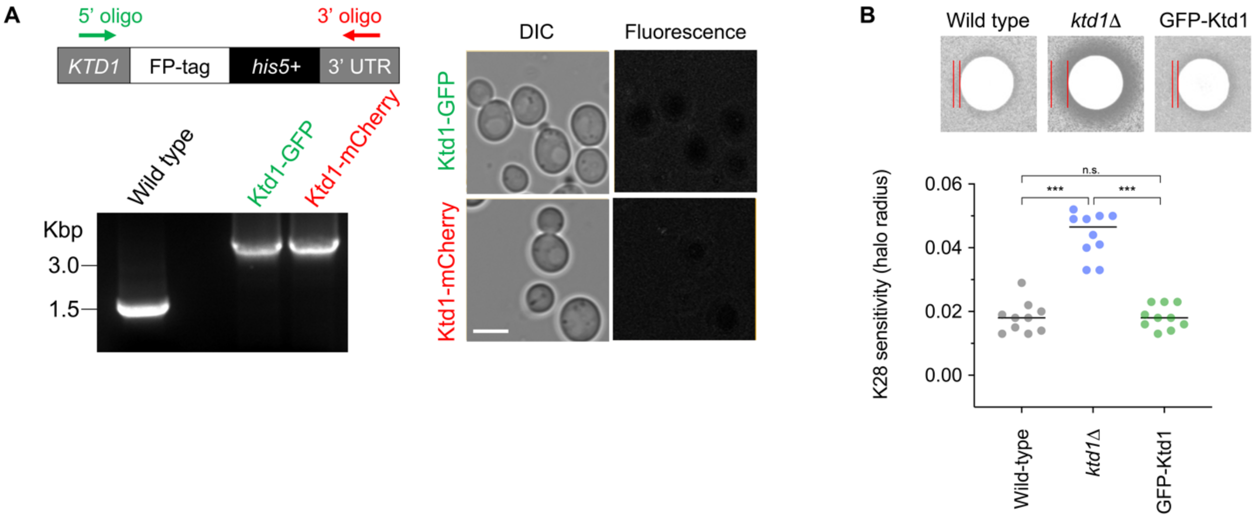

Supplementary Figure 9. Fluorescent tagging of Ktd1p for localization studies.
A, Genotyping strategy to confirm fluorescent protein sequence and *his5*$^{+}$ selection cassette successfully integrated at *KTD1* locus (left) and Airyscan2 imaging showing failure to detect any fluorescent Ktd1p fusion proteins (right). **B,** GFP-Ktd1p confers K28 resistance. Three colonies of each indicated strain were grown to mid-log phase before 1 mL of culture at $OD_{600} = 0.25$ was harvested, washed twice with water, and then used to plate a lawn on SC media containing agar. Following drying for 30 minutes, 7.5 µL of a hypersecretor strain MSY52 grown to mid-log phase, separately was spotted on each lawn for a total of 10 spots per genotype, dried, and yeast growth recorded after 48 hours growth at 30C. The radii of 'halo' zones of inhibition (red lines) were measured using ImageJ. Bottom panel shows 10 radii determined per genotype, with the horizontal black line delineating the genotype median. (*** $P < 0.0001$ by two-sample t-test.)

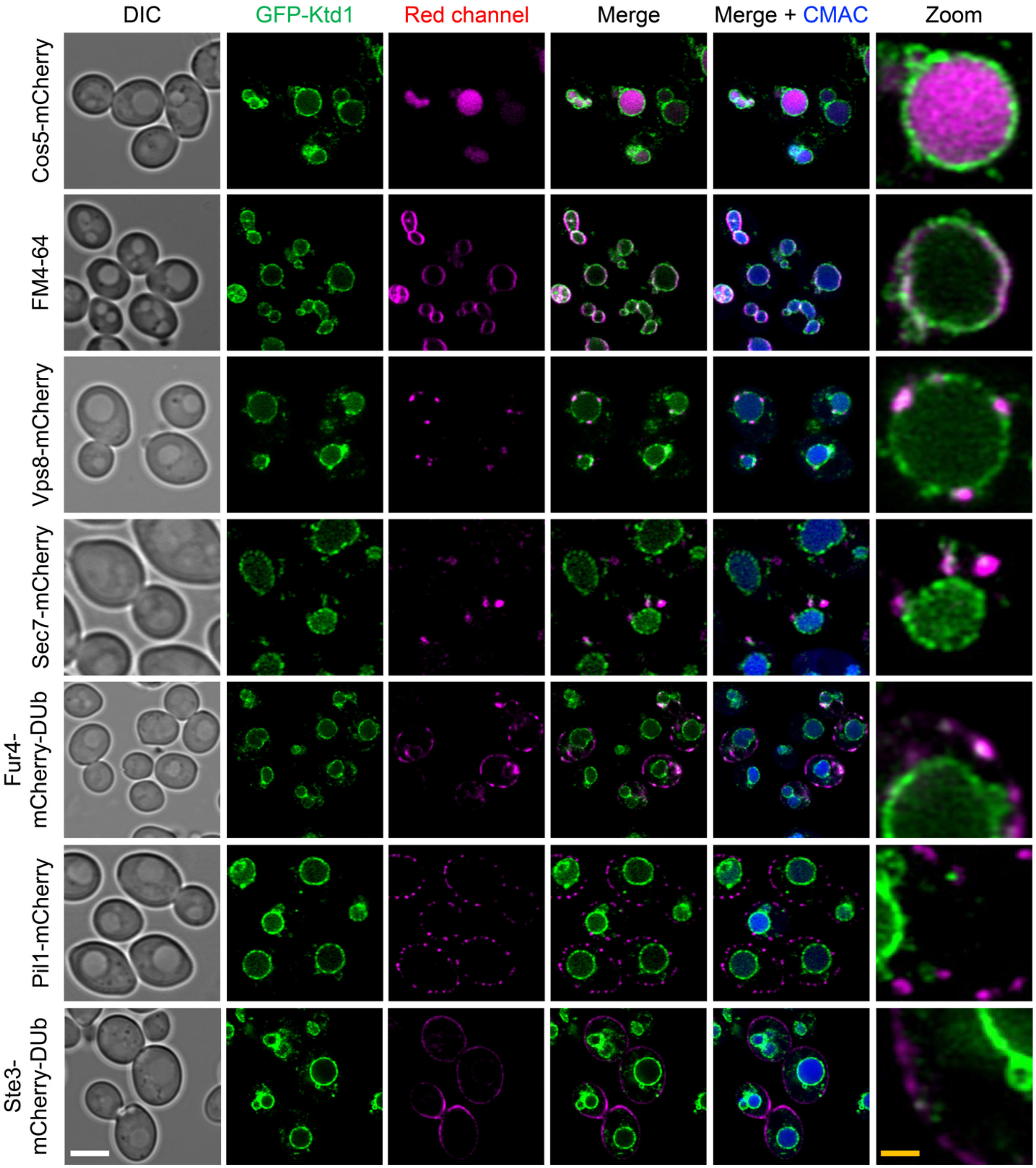

Supplementary Figure 10. Localization of Ktd1p by confocal microscopy.
Indicated red fluorescent markers were imaged in cells also expressing GFP-Ktd1p and labeled for 30 minutes with CMAC minimal media prior to imaging. White scale bar, 5μm and orange scale bar, 1μm.

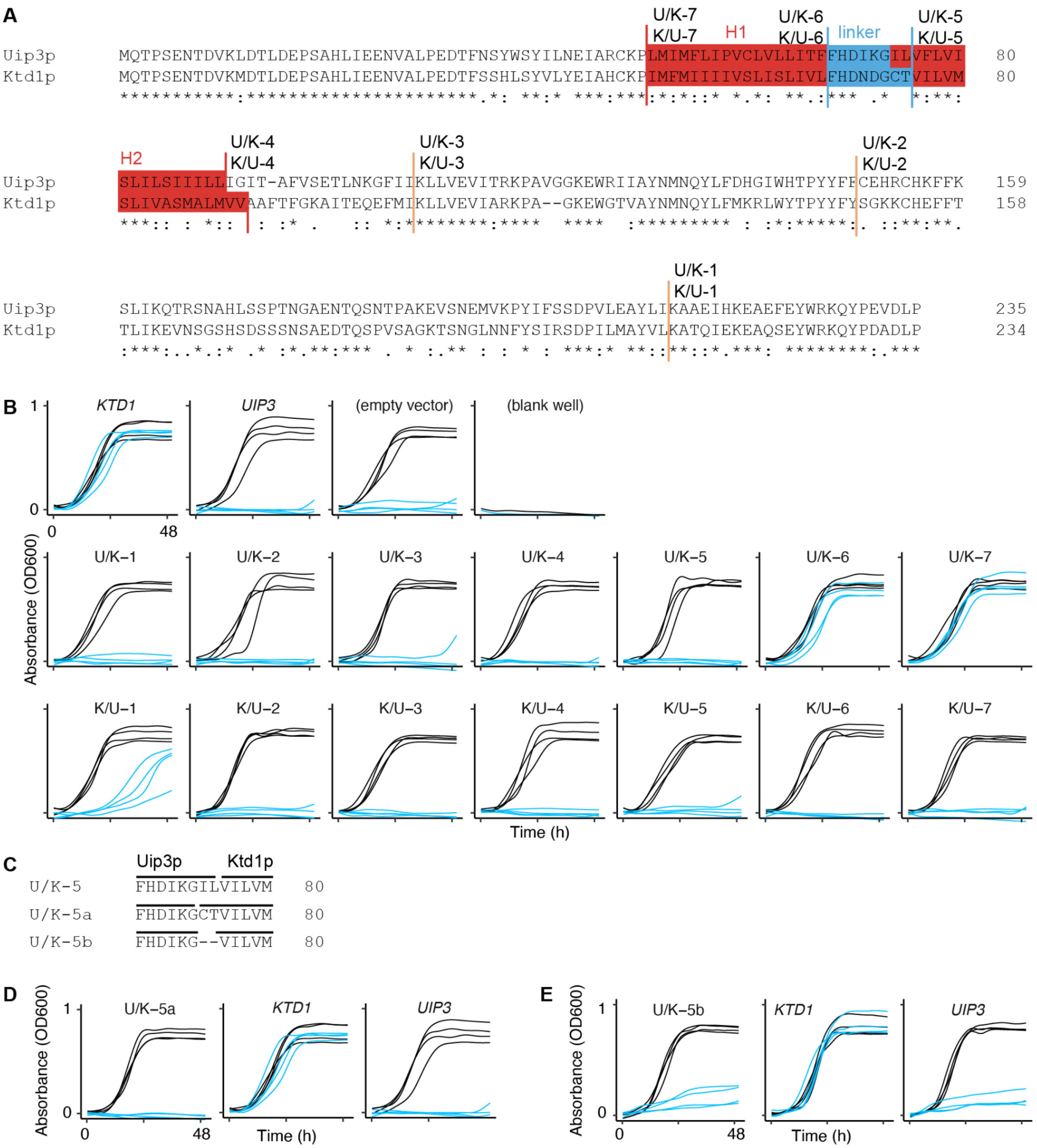

Supplementary Figure 11. Analysis of chimeras between Uip3p and Ktd1p.
A, Alignment of Ktd1p and Uip3p. Chimera transition points are marked with vertical lines and the names of the chimeras transitioning there. The transmembrane helices H1 and H2 are colored in red and the linker between them is colored in blue. We used the positions of the transmembrane domains as defined by Poirey et al. (Poirey *et al.* 2002). **B,** The K28 resistance shown in Fig. 5A was quantified as AUC_{+K28} / AUC_{-K28} calculated from the growth curves shown here of BY *ktd1Δ* expressing the labeled chimeras, with *n* = 4 biological replicates per strain. Blue growth curves are of strains grown in media containing K28, and black growth

curves are of strains grown in media lacking K28. **C,** We additionally tested two alternative Uip3p-Ktd1p chimera transition points near the transition point used in U/K-5. U/K-5a transitions at a conserved glycine within the interhelix linker, and U/K-5b transitions from the end of Uip3p's linker to the start of Ktd1p's H2 transmembrane domain. **D,** Growth curves of BY *ktd1Δ* expressing U/K-5a in media with and without K28. BY *ktd1Δ* expressing either *KTD1* or *UIP3* are shown for comparison. **E,** Growth curves of BY *ktd1Δ* expressing U/K-5b in media with and without K28. BY *ktd1Δ* expressing either *KTD1* or *UIP3* are shown for comparison. In D and E, U/K-5a and U/K-5b conferred significantly less resistance than *KTD1* ($P < 0.001$ by Welch's two-sample *t*-test) and were not distinguishable from *UIP3*. U/K-5a was tested in the same experiment as the chimeras shown in B; U/K-5b was tested separately.

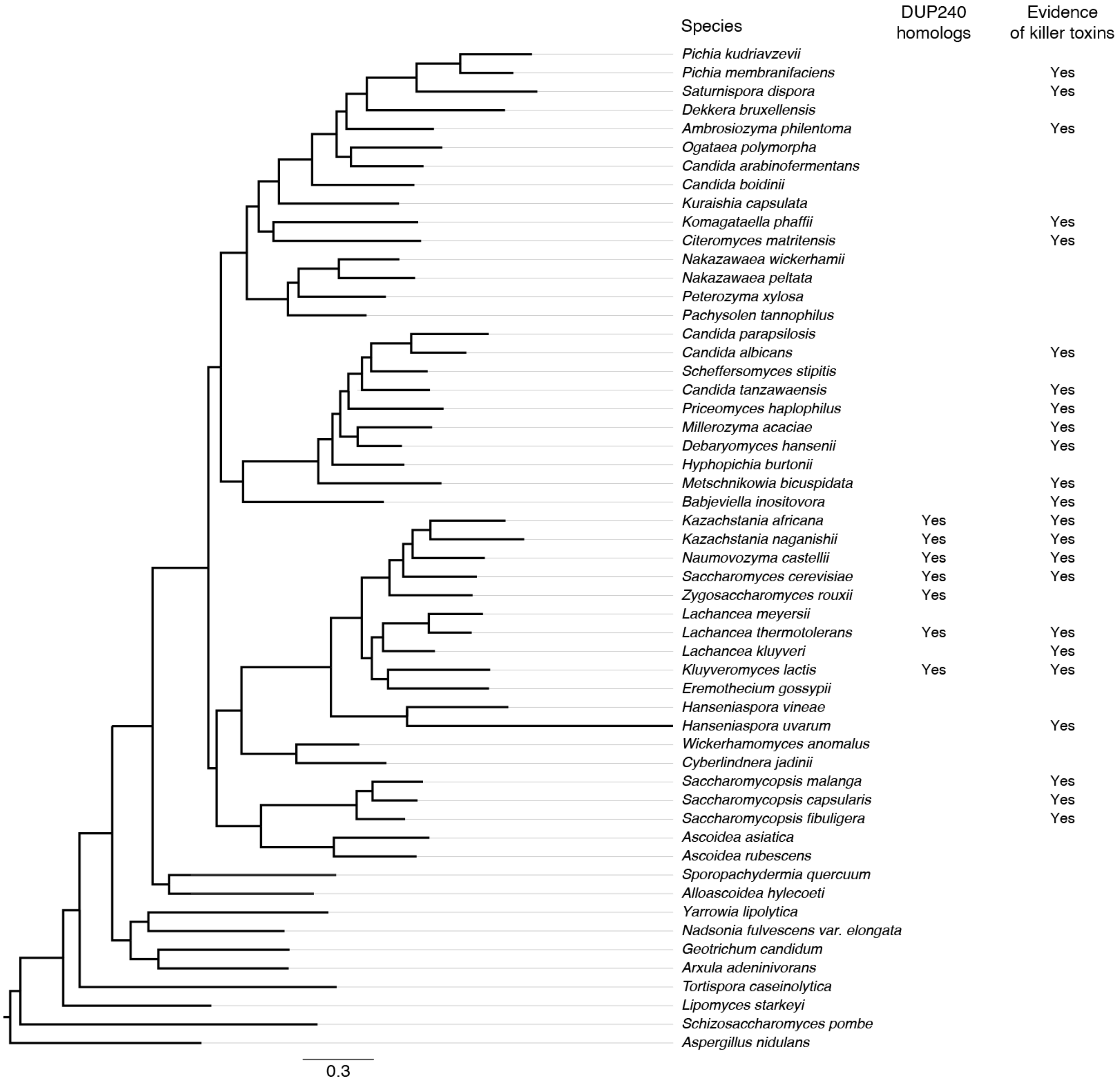

Supplementary Figure 12. Species distribution of homologs of DUP240 genes.
Homologs of DUP240 genes were found by performing tblastn searches against each genome shown on the tree, with the Ktd1p protein sequence as query. All negative hits were negative both with the default tblastn search parameters and with the tblastn Expect threshold relaxed to 0.5. Evidence of killer toxins is largely as reported by Krassowski et al. (Krassowski *et al.* 2018), with the exception of *P. membranifaciens, K. africana, N. castellii,* and *H. uvarum* (Zorg *et al.* 1988; Belda *et al.* 2017; Fredericks *et al.* 2021). Phylogenetic species tree modified with permission from Krassowski et al.

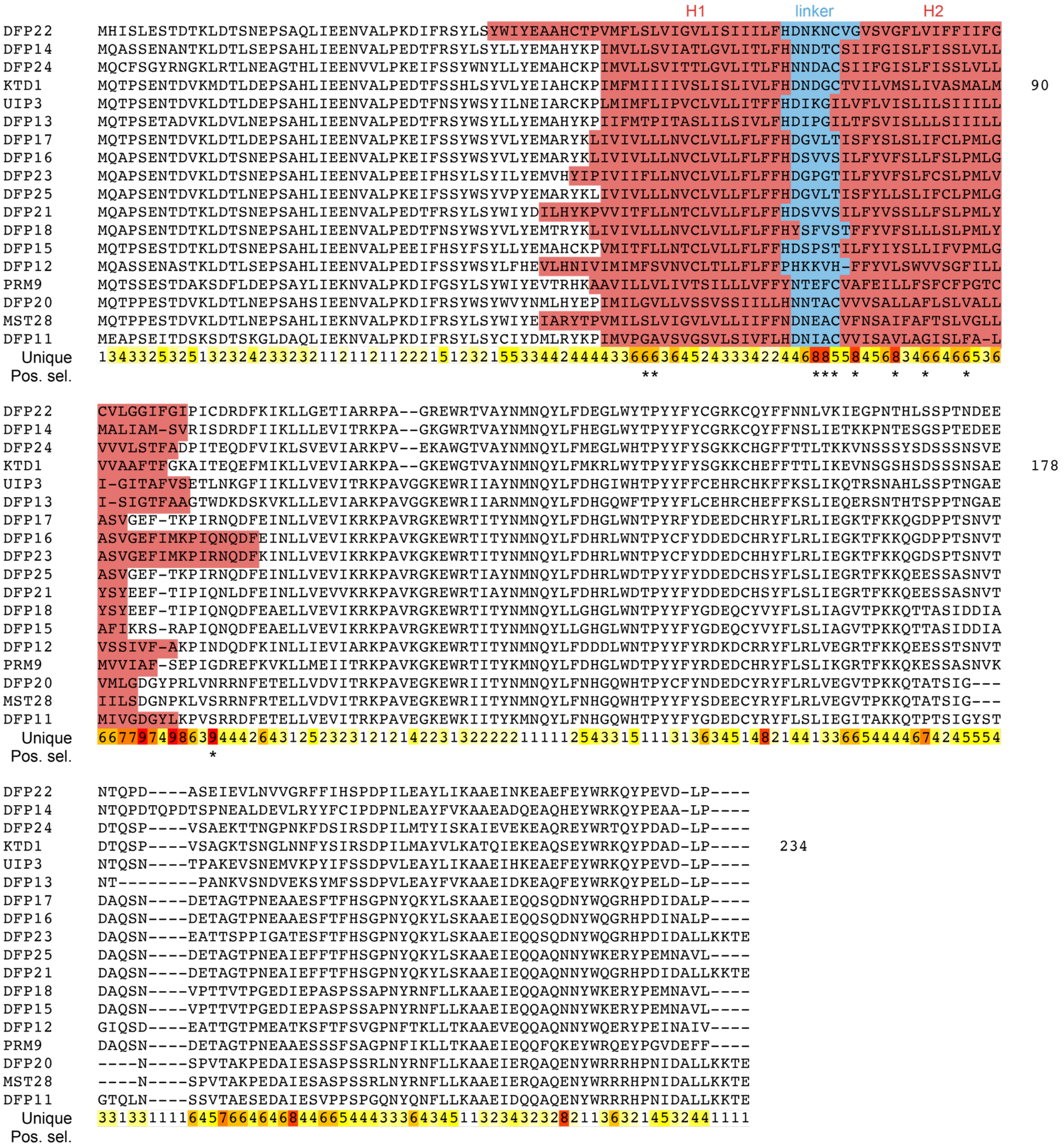

Supplementary Figure 13. Regions of divergence between DUP240 proteins.
In our 16-isolate panel we found 25 DUP240 genes; of these, the 18 full-length DUP240 proteins most similar to Ktd1p are shown in this alignment. DUP240 genes with no previous standard name were named DFP (DUP240 family protein). The locations of transmembrane helices H1 and H2 were determined by PHOBIUS (Käll *et al.* 2004). Along the bottom of the alignment, we show the number of unique amino acids represented at each position in the alignment ("Unique") and the sites identified as experiencing positive selection ("Pos. sel."); see Fig. 5B.

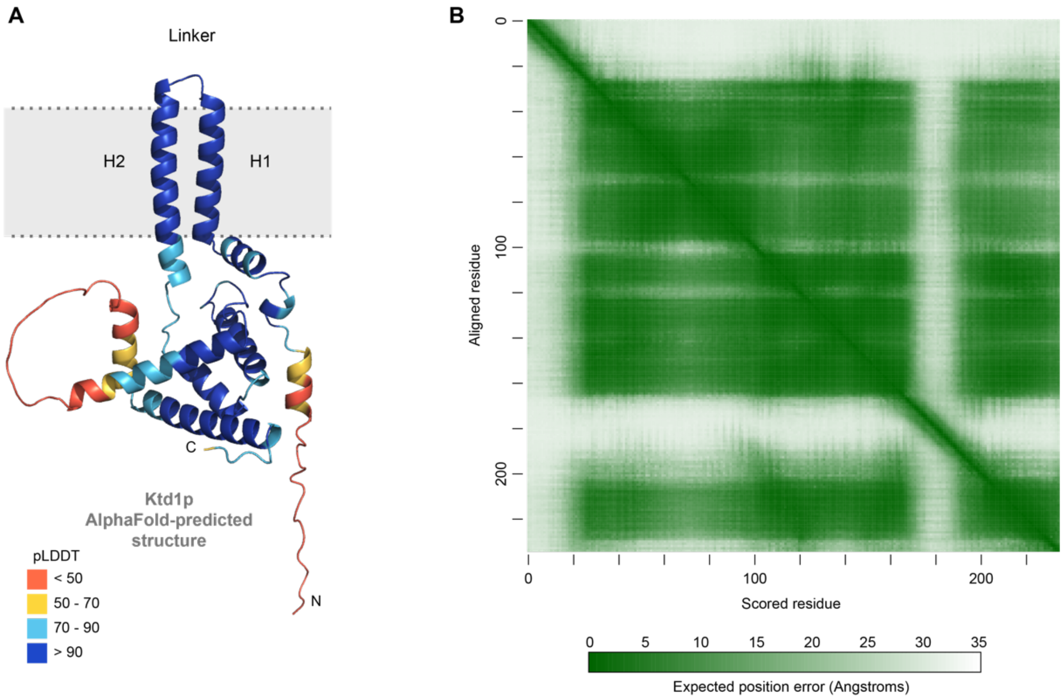

Supplementary Figure 14. AlphaFold model confidence.
A, pLDDT-colored structure of Ktd1p. The predicted local distance difference test (pLDDT) is a per-residue confidence score of an AlphaFold prediction that estimates how well the prediction would agree with an experimental structure (Jumper *et al.* 2021), with higher values corresponding to higher confidence. **B,** Predicted aligned error matrix. The values are estimates of the error in the modeled position of a scored residue relative to a given aligned residue.

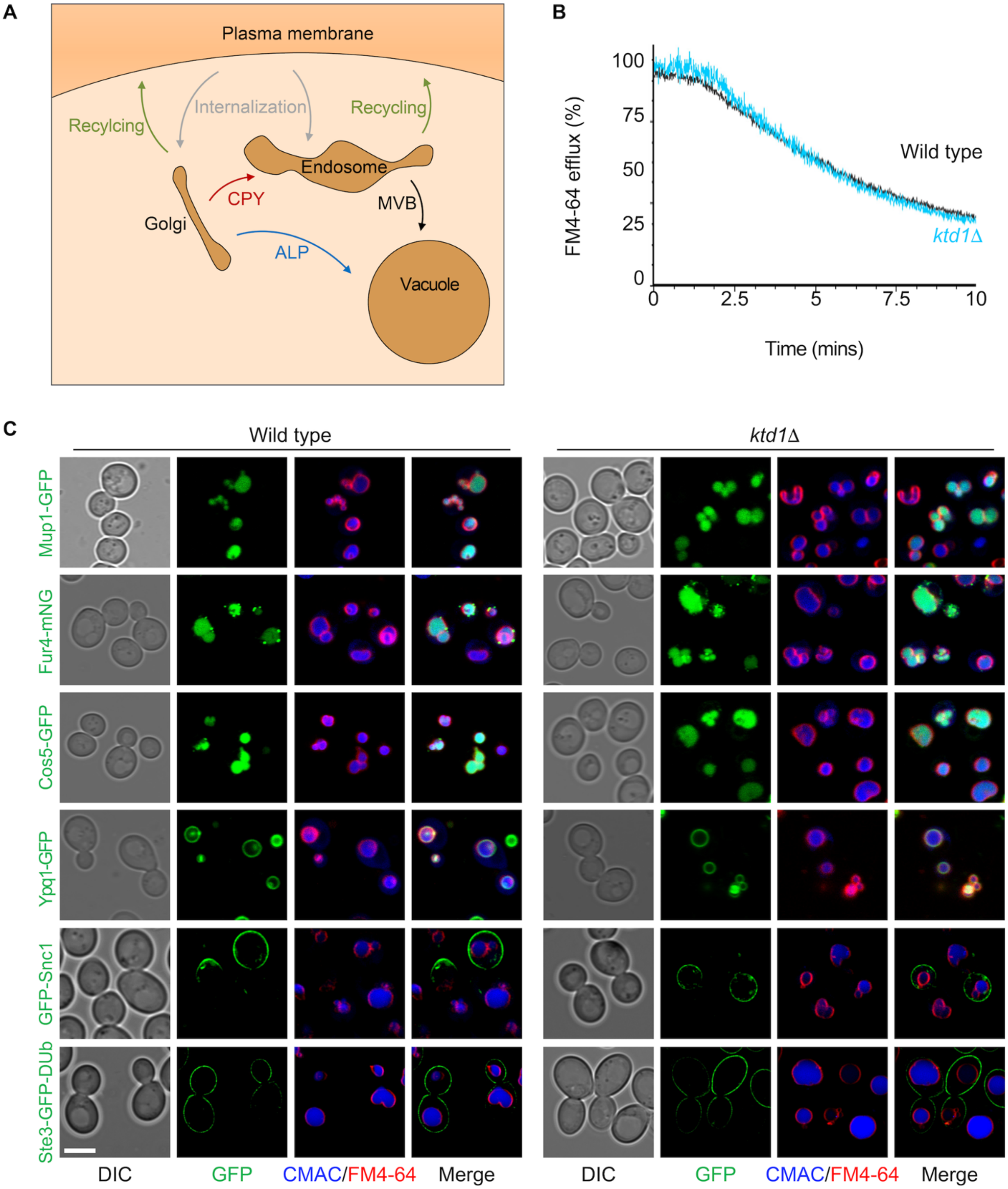

Supplementary Figure 15. Cargo sorting in *ktd1Δ* mutants.
A, Schematic diagram showing the main membrane trafficking routes of the yeast endolysosomal system. This includes recycling pathways of internalized material via the Golgi and endosome (used by Snc1, Ste3-GFP-DUb and FM4-64), the MVB sorting of surface proteins to the vacuolar lumen (used by Fur4 and Mup1), MVB sorting of CPY directly from the Golgi (used by Cos proteins) and the ALP pathway from the Golgi that bypasses the MVB (used by Ypq1). **B,** FM4-64 was loaded to the endosomes of wild-type (black) and *ktd1*Δ mutants (blue)

for 8 minutes in YPD at room temperature followed by 3 x 5min washes in ice cold SC media. Cells were then resuspended room temperature SC media and fluorescence measured (approximately 1 million cells / minute) for 10 minutes. White scale bar, 5µm.

Strain Name	Environmental / Geographical Origin
RM11-1a (MATalpha)	Zinfandel vineyard / CA, USA
M22 (MATalpha)	Vineyard / Italy
YJM981 (MATalpha)	Vagina / Italy
I14_1b (MATa)	Vineyard soil / Italy
CLIB413_1b (MATa)	Fermenting rice / China
273614N (MATa)	NA / NA
PW5_b (MATalpha)	Raphia palm wine / Nigeria
BY (MATa)	(Prototrophic version of a common lab strain)
YJM454 (MATa)	Human, clinical / NA
YJM145 (MATalpha)	AIDS patient / NA
Y10 (MATalpha)	Coconut / Philippines
CLIB219 (MATalpha)	Wine / Russia
YPS1009 (MATalpha)	Exudate, *Quercus* sp. (oak) / NA
CBS2888 (MATa)	Soil / South Africa
YPS163 (MATa)	Soil beneath *Quercus rubra* (oak) / PA, USA
YJM978 (MATalpha)	NA / NA

Supplementary Table 1. Origin of the *S. cerevisiae* strains in the 16-isolate panel surveyed in this study
Strains and their origin information (Peter *et al.* 2018; Bloom *et al.* 2019). NA = information not available.

QTL	Chr	Peak Marker Position	Left Marker	Right Marker	LOD Score
1	I	184648	180646	184686	49.72978
2	XII	745464	250697	967302	4.11199
3	XIV	468488	467028	485549	11.36227

Supplementary Table 2. QTLs identified from linkage mapping of K28 resistance variation in a BY × RM cross.

912 segregants from a BY × RM cross were phenotyped for resistance to K28 killer toxin, measured as AUC_{+K28}/AUC_{-K28}. The genotypes at 28,220 biallelic markers spanning the genome were associated with K28 resistance, generating the LOD plot in Fig. 2B. We identified three QTLs that passed a 5% family-wise error rate (FWER) LOD threshold of 3.55, computed from 1000 iterations of randomly assigning phenotypes to segregants and calculating the top LOD score. The left and right markers denote 95% confidence intervals for the position of the LOD peak marker. These were determined from the positions of peak LOD scores on chromosomes I, XII, and XIV from 1000 bootstrap samplings.

Gene ID	Gene name	What is known	Reference
YAR023C	*DFP1*	Premature stop in reference allele	(Wirth *et al.* 2005)
YAR027W	*UIP3*	Interacts with Ulp1p	(Takahashi *et al.* 2000)
YAR028W	*KTD1*	Protects against K28	This study
YAR029W	*DFP2*	Pseudogene	(Wirth *et al.* 2005)
YAR031W	*PRM9*	Interacts with COPII	(Sandmann *et al.* 2003)
YAR033W	*MST28*	Interacts with COPI	(Sandmann *et al.* 2003)
YCR007C	*DFP3*		
YGL051W	*MST27*	Interacts with COPI; multicopy suppressor of COPI mutants	(Sandmann *et al.* 2003)
YGL053W	*PRM8*	Interacts with COPII	(Sandmann *et al.* 2003)
YHL044W	*DFP4*		

Supplementary Table 3. DUP240 genes in the yeast reference genome.
We have named the four remaining unnamed DUP240 genes in the *S. cerevisiae* reference genome as *DFP* (DUP240 family proteins).

**Segment (*KTD1* a.a.)**	**Mean dN/dS (Site model)**	**Likelihood-ratio test statistic 2*[ln(L_{M8})-ln(L_{M7})]**	***p*-value of chi² test (df = 2)**	***KTD1* sites under positive selection with Pr(dN/dS>1) using BEB (* > 0.95, ** > 0.99)**
**1-18**	0.9036 (M7)	1.77	0.413	N/A
**19-36**	0.2911 (M7)	0.90	0.637	N/A
**37-47**	0.4695 (M7)	0.62	0.733	N/A
**48-89**	2.0006 (M8)	40.28	**1.795×10^{-9}**	55I, 0.986 (*)<br>56I, 0.991 (**)<br>72D, 1.000 (**)<br>73G, 0.988 (*)<br>74C, 0.973 (*)<br>76V, 0.997 (**)<br>80M, 0.992 (**)<br>83I, 0.999 (**)<br>87M, 0.970 (*)
**90-99**	1.0000 (M7)	3.61	0.165	N/A
**100-124**	0.6415 (M8)	7.66	**0.021**	102T, 0.998 (**)
**125-210**	0.5975 (M7)	2.45	0.294	N/A
**211-234**	0.4283 (M7)	0.17	0.919	N/A

Supplementary Table 4. Analysis of rapidly evolving sites in the DUP240 family.
Sites under positive selection were identified using the codeML module in PAML. The DUP240 homolog alignment (shown in Fig. S11) was divided into segments according to results of DNA recombination breakpoint analysis using GARD. Model M7 (negative or neutral selection) and Model M8 (negative, neutral, or positive selection) were compared using the likelihood-ratio test, followed by the chi-squared significance test to determine where Model M8 should be accepted, which would indicate the presence of a signature of positive selection in the region. Segments 48-89 and 100-124 were identified as having such signatures. Bayes Empirical Bayes (BEB) analysis on these segments revealed sites under positive selection (dN/dS > 1) with posterior probability Pr(dN/dS > 1) greater than 0.95. Codon positions are with respect to *KTD1*.

Supplementary Dataset 1. Loss-of-function mutations in genes that affect K28 resistance. Loss-of-function mutations were identified across the 16-isolate panel in any of the 346 non-dubious genes identified by Carroll et al. as affecting K28 sensitivity when deleted in BY. Mutations with consequence "frameshift" or "nonsense" were identified using the *variantAnnotation* package in R from the genetic variants found by Bloom et al. Variants were filtered for those that were at least 10 codons from the gene end and which were observed in Pacbio sequencing of these genomes. The table shows the effect of the gene knockout on K28 sensitivity as determined by Carroll et al. (Very hypersensitive = 3-5 mm halo, partially hypersensitive = 2.5-3 mm halo, WT = 2 mm halo, partially resistant = 1-1.5 mm halo, very resistant = 0-1 mm halo. Halo size was not specified for strains annotated as hypersensitive.) (Included in a separate excel file.)

Supplementary Dataset 2. DUP240 homologs. A, Presence and absence of DUP240 homologs in the genomes of strains in the 16-isolate panel. The genomes of the 16-isolate panel were iteratively searched using BLASTn to find DUP240 ORFs, starting from the 10 DUP240 genes from the reference genome. This process identified 16 non-reference DUP240 genes, which we designated *DFP11-DFP26*. Alleles labelled with an asterisk (*) have premature stop codons or frameshift mutations predicted to render them nonfunctional. The sequences of the alleles marked in boxes were treated as the reference alleles in all sequence analyses. **B,** Detailed DUP240 homolog information. Information is provided on each DUP240 homolog identified in the 16 genomes. The homologs are named by the DFP gene they were most similar to. The information provided includes the chromosome, strand, start and end position for where the homolog was found, as well as the homolog's length, whether it is intact, whether it is within 30 kb of the chromosome end, and its sequence. Note that two DUP240 homologs are listed as being on "chrXVItrunc," which is the product of a reciprocal translocation between chrVIII (on which DFP4 (YHL044W) is found in the reference genome) and chrXVI. (Included in a separate excel file.)

Supplementary Dataset 3. Strains, plasmids, and oligonucleotides. A, Strains used in this study. **B,** Plasmids used in this study. **C,** Oligonucleotides used in this study. (Included in a separate excel file.)
